# Reproductive tissue–derived stromal cells rescue fertility by coupling follicular activation with endometrial remodeling

**DOI:** 10.1093/stcltm/szag013

**Published:** 2026-03-27

**Authors:** Veronika Viktorija Borutinskaitė, Indrė Krastinaitė, Elvina Valatkaitė, Aistė Zentelytė-Vilkė, Rūta Navakauskienė

**Affiliations:** Department of Molecular Cell Biology, Institute of Biochemistry, Life Sciences Center, Vilnius University, Vilnius, LT-01257, Lithuania; Department of Molecular Cell Biology, Institute of Biochemistry, Life Sciences Center, Vilnius University, Vilnius, LT-01257, Lithuania; Department of Molecular Cell Biology, Institute of Biochemistry, Life Sciences Center, Vilnius University, Vilnius, LT-01257, Lithuania; Department of Molecular Cell Biology, Institute of Biochemistry, Life Sciences Center, Vilnius University, Vilnius, LT-01257, Lithuania; Department of Molecular Cell Biology, Institute of Biochemistry, Life Sciences Center, Vilnius University, Vilnius, LT-01257, Lithuania

**Keywords:** endometrium, follicular fluid, placenta, menstrual blood, stromal cells, regeneration, infertility

## Abstract

**Background:**

Mesenchymal stromal cells (MSCs) of various origins promote regeneration through paracrine signaling, immune modulation, and angiogenesis support. Premature ovarian failure (POF) is an excellent model to study coordinated ovarian and uterine repair, as cytotoxic injury simultaneously depletes ovarian follicles and impairs uterine receptors, resulting in infertility.

**Methods:**

We established a busulfan/cyclophosphamide (Bu/Cy) POF model and applied human MSCs derived from reproductive/perinatal tissues—endometrium (hEndSCs), menstrual blood (hMenSCs), placenta (hPSCs), or follicular fluid (hFFSCs)—to treat the condition. The primary endpoint was pregnancy rate; secondary endpoints included serum anti-Mullerian hormone (AMH) levels and ovarian/uterine molecular profiles (RT-qPCR panels; selected ovarian signaling proteins by Western blot).

**Results:**

Chemotherapy reduced fertility (0%) and AMH levels compared to healthy controls. MSC therapy restored fertility in 41%–75% of mice, with hEndSC and hMenSC achieving the highest pregnancy rates (both 75%) and the highest AMH recovery. In the ovary, MSC increased *Amh, Gdf3, Gja1, Zp1*, and, depending on the source, Fshr, with concomitant activation of PI3K/AKT/mTOR effectors (p-AKT, p-mTOR, p-GSK3β, p-PDK1). In the uterus, MSC increased the expression of *Col1a1, Col3a1, Ctgf, Pcna, Ccnd1*, and *Ki6*7, consistent with extracellular matrix repair and proliferative renewal.

**Conclusions:**

These data suggest that MSCs derived from reproductive tissues, particularly endometrial origin, may restore fertility in POI by linking follicular activation to endometrial remodeling and support the translational development of MSC therapies that address both follicular depletion and uterine competence in infertility.

## Graphical abstract

**Graphical Abstract:**
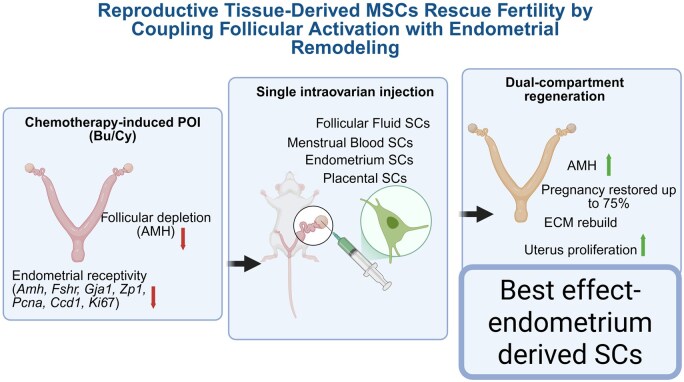

Significance statement Premature ovarian insufficiency after chemotherapy leaves many patients without a realistic path back to biological motherhood because current options rarely repair the ovary and uterus at the same time. Here, we show that mesenchymal stromal cells from reproductive tissues can bridge that gap: a single intraovarian dose restored pregnancies while partially rebuilding endocrine output and uterine readiness. The benefit was not the same across stromal cell sources—cells from endometrium and menstrual blood were the most effective—pointing to practical guidance for future clinical use. Mechanistically, ovarian markers of follicular activation and PI3K/AKT/mTOR signaling rose in step with uterine programs for matrix repair and proliferation, tying functional recovery to coherent tissue biology. Together, these findings position reproductive tissue–derived MSCs as a credible, translatable strategy for infertility care, aimed at restoring both follicles and an implantation-ready endometrium rather than treating them in isolation.

## Introduction

Human mesenchymal stromal cells (hMSCs)—due to their ability to self-renew, differentiate and, most importantly, transmit trophic paracrine signals—are widely studied for tissue repair. hMSCs support cell survival, proliferation, angiogenesis, and immunomodulation and are therefore already being implemented in the therapies of cardiovascular and degenerative diseases.^1^^,^^2^ In recent years, there has been increasing interest in their application in female infertility, but mechanistic insights in the field of reproduction are still lacking, and direct comparisons of hMSCs of different human tissue origin in assessing fertility restoration are practically nonexistent.^3^

Infertility affects ∼12% of families worldwide.^4^ Among the most important causes is premature ovarian insufficiency (POF/POI), which is detected in up to 4% of women.^3^^,^^5^ POF is characterized by ovarian dysfunction, loss of follicular reserve, and hormonal imbalance, causing menopausal symptoms and long-term consequences such as osteoporosis, heart and joint diseases, and mental health disorders.^6–9^ The etiology is diverse: genetic, autoimmune, infectious, as well as iatrogenic—especially after chemo- and radiotherapy.^10^^,^^11^ Young cancer patients are at particularly high risk because many chemotherapeutic agents are gonadotoxic, accelerating follicular “burnout,” inducing follicular apoptosis or damaging ovarian tissue.^8^ Current measures, including hormone replacement therapy, alleviate endocrine deficits but do not restore ovarian function or uterine receptivity.^7^ Since POF simultaneously damages the ovaries and uterus, effective fertility restoration is only possible through coordinated regeneration of both compartments.

Here, we explicitly test whether tissue of origin matters for reproductive repair. Using a standardized, severe busulfan/cyclophosphamide POF model, we compared 4 reproductive/perinatal hMSC sources—endometrium (hEndSCs), menstrual blood (hMenSCs), placenta (hPSCs), and follicular fluid (hFFSCs)—delivered under identical dose, route, and timing. Our primary endpoint was functional fertility (pregnancy rate and embryo counts), with secondary readouts capturing coordinated ovarian–uterine recovery (serum AMH; ovarian folliculogenesis markers and PI3K/AKT/mTOR signaling; uterine extracellular-matrix remodeling and proliferation programs). We hypothesized that source-specific paracrine profiles differentially couple follicular activation with endometrial remodeling, yielding distinct efficacy signatures across MSC types.

## Methods

### Isolation and cultivation of human mesenchymal stromal cells

#### Donor material, consent, and ethics

Human tissues/fluids (endometrium, follicular fluid, menstrual blood, placenta) were obtained with written informed consent under approval of the Vilnius Regional Biomedical Research Ethics Committee (No. 158200-18/7-1049-550). Samples were anonymized prior to processing.

#### General culture conditions (applies to all hMSC sources unless stated)

Cells were expanded in DMEM/F-12 (Corning, USA) supplemented with 10% fetal bovine serum (FBS; Capricorn Scientific, Germany), 100 U/mL penicillin, and 100 μg/mL streptomycin (Sigma-Aldrich, USA). Cultures were maintained at 37 °C, 5% CO_2_, and 95% relative humidity. All isolations were performed under sterile conditions. After initial plating, nonadherent cells were removed by medium change per source-specific protocols cited above. Adherent cells were expanded under the common culture conditions detailed above.

#### Endometrium-derived MSCs (hEndSCs): isolation followed Tavakol et al. and Valatkaite et al. with minor adaptations

Briefly, endometrial tissue was minced mechanically into ∼1–2 mm fragments, incubated with collagenase I (1 mg/mL; STEMCELL Technologies, Canada) at 37 °C for 30–45 min and then filtered to remove debris. The cell suspension was washed with PBS, pelleted, and resuspended in culture medium for plating.^12^^,^^13^

#### Follicular-fluid stromal cells (hFFSCs): isolation followed Skliutė et al.

Follicular fluid was collected at oocyte retrieval prior to ART procedures and centrifuged at 500×*g* for 10 min. The supernatant was discarded. Red blood cells were lysed using a 10× stock buffer (final working components: 155 mM NH_4_Cl, 12 mM NaHCO_3_, 0.1 mM EDTA; pH 7.3; Sigma-Aldrich, USA). Cells were then washed with PBS, pelleted, and resuspended in growth medium for culture.^14^

#### Menstrual blood–derived MSCs (hMenSCs): isolation followed Skliutė et al.

Fresh menstrual blood was incubated with collagenase II (50 U/mL; Sigma-Aldrich, USA) for 20 min at 37 °C, 5% CO_2_. Mononuclear cells were then isolated using Ficoll-Paque™ PREMIUM per manufacturer’s protocol, washed with PBS, and plated in culture medium.^15^

#### Placenta-derived MSCs (hPSCs): isolation followed Navakauskienė et al. and Shablii et al.

Placental tissue (post-cesarean section) was washed in Hank’s balanced salt solution containing 100 U/mL penicillin and 50 mg/mL streptomycin, minced into ∼1–3 mm pieces, and digested in DMEM (Corning, USA) with 0.1% collagenase I, 0.6 U/mL dispase I (STEMCELL Technologies, Canada), and HEPES buffer (SERVA Electrophoresis GmbH, Germany). The enzymatically and mechanically dissociated suspension was filtered as needed, washed, and transferred to growth medium for culture.^16^^,^^17^

### Giemsa staining

The MSCs were fixed for 3 min in 100% methanol. The fixed MSCs were stained for 40–60 min with Giemsa solution (dye diluted in PBS solution 1:9; Sigma-Aldrich, USA), washed 5–8 times with running water. The images of MSCs were taken with an optical inverted microscope Motic AE2000 (Motic, China).

### Flow cytometry

For flow cytometry analysis, primary hMSCs are washed twice in buffer (1X PBS/1% bovine serum albumin [BSA]) and centrifuged at 500*g* for 5 min +4 °C. Cells were resuspended in 50 μL PBS with 1% BSA solution, and specific antibodies were added to 0.05 ×·10^6^ cells and incubated 30 min in the dark at 4 °C. The following antibodies were used: FITC conjugated anti-Hu CD73 (1F-675-T100), Anti-Hu CD44 (1F-221-T100), Anti-Hu CD29 (1F-219-T100) from Exbio (Czech Republic), and anti-CD34 (343604) from Biolegend (USA); APC-conjugated Anti-Hu CD90 (1A-652-T100) and Anti-Hu CD105 (1A-298-T100) from Exbio (Czech Republic). Isotype controls were used to calculate the percentage of positive cells in the population (%), following the manufacturer’s recommended protocol. Cells were washed twice in PBS + 1% BSA and finally analyzed using a Guava^®^ easyCyte™ 8HT flow cytometer. Results were processed with the Flowing Software 2 software.

### RNA isolation and RT-qPCR

Total RNA was extracted from hMSC and mouse uterus and ovary tissues using the Quick-DNA/RNA™ Miniprep Kit (Zymo Research, USA), and cDNA was synthesized with the LunaScript^®^ RT SuperMix Kit (New England Biolabs, USA) following the manufacturer’s recommendations. RT-qPCR was performed using Luna^®^ Universal qPCR Master Mix (New England Biolabs, USA) and Rotor-Gene 6000 thermocycler with Rotor-Gene 6000 series software (Corbett Life Science, QIAGEN). Gene expression was normalized to *GAPDH* or *Actb* and *18 s*, and relative gene expression was calculated using the ΔΔCt method. The following primers were used in the study: *Actb*: forward (F) – TGTTACCAACTGGGACGACA, reverse (R) – CCATCACAATGCCTGTGGTA; *Amh*: F– CAGTTGCTAGTCCTACATC, R–TCATCCGCGTGAAACAGCG; *Amhr2*: F– GTATCCGCTGCCTCTACAGC, R–AGCCTGGCTCATCACTGTCT; *Ccnd1*: F–GCGTACCCTGACACCAATCTC, R–CTCCTCTTCGCACTTCTGCT; *Col1a1*: F–TCACCAAACTCAGAAGATGTAGGA, R–GACCAGGAGGACCAGGAAG; *Col3a1*: F–ACAGCAGTCCAACGTAGATGAAT, R–TCACAGATTATGTCATCGCA; *Ctgf*: F–GGACACCTAAAATCGCCAAGC, R–ACTTAGCCCTGTATGTCTTCACA; *Cxcl15*: F–TGTTGAGCATGAAAAGCCTCTAT, R–AGGTCTCCCGAATTGGAAAGG; *Ddx4*: F–GAGAACACATCTACAACTGGTGG, R–CCTCGCTTGGAAAACCCTCT; *Foxa2*: F–GGAGGCAAGAAGACCGCTC, R–CCTTTAGCTCGCTTAGGCCAC; *Fshr*: F—AGTTGCATGGCATGTGTGAT, R–CATCACTGGGAACACCACG; *Gdf3*: F–ACCTTTCCAAGATGGCTCCT, R–CCTGAACCACAGACAGAGCA; *Ki67*: F–ATCATTGACCGCTCCTTTAGGT, R–GCTCGCCTTGATGGTTCCT; *Mcam*: F–ACCGCCTTAGCCTCCAAGA, R–AGGAACATTCGCTCATCATGG; *Pdgfrb*: F–AGGAGTGATACCAGCTTTAGTCC, R–CCGAGCAGGTCAGAACAAAGG; *Pcna*: F–TTGCACGTATATGCCGAGACC, R–GGTGAACAGGCTCATTCATCTCT; *Zp1*: F–CCCTGAGATTGGGTCAGCG, R–AGAGCAGTTATTCACCTCAAACC; *18 s*: F–GGAAGGGCACCACCAGGAGT, R–TGCAGCCCCGGACATCTAAG.

### Protein purification and Western blot analysis

The total protein from the ovaries and uterus was extracted by precipitation of acetone. Mice organs were submerged in liquid nitrogen and, using a mortar and a pestle, ground until a powder consistency was reached. After this, 600 µL of DNA/RNA lysis buffer was mixed with the powder, homogenized, and then transferred to the Zymo-Spin™ Column followed by DNA and RNA isolation using the Quick-DNA/RNA™ Miniprep Kit (Zymo research, USA). After binding of RNA to the column, the protein content in the flow-through was purified by adding 4 volumes of cold acetone (−20 °C). The samples were incubated at −20 °C overnight and then centrifuged at 8000×*g* for 10 min at 4 °C. The supernatant was discarded and the pellet was washed twice with 80% acetone by centrifugation at 8000 ×g for 5 min at 4 °C. The protein pellet was then air-dried at room temperature and resuspended in RIPA buffer (150 mM NaCl, 10 mM EDTA pH 8, 10 mM Tris pH 7.4, 0.1% SDS, 1% deoxycholate, 1% NP-40 in 1X PBS pH 7.6). After measuring protein concentration, SDS lysis buffer (125 mM Tris, pH 6.8, 4% SDS, 200 mM DTT, 20% glycerol and traces of bromophenol blue) was added to protein samples. The samples were further fractionated in gel electrophoresis with 7.5%–15% gradient polyacrylamide SDS-PAGE and Western blot analysis was performed. Separated proteins were transferred onto PVDF membrane, which was then incubated with blocking solution (4% milk in PBS with 0.05% Tween [PBST-0.05%]/5% BSA in Tris-buffered saline with 0.05% Tween [TBST-0.05%] solution) for 2 h and with primary antibodies overnight at 4 °C. Primary antibodies were used to detect the following targets: β-tubulin (dilution 1:500), Akt (1:1000), phospho-Akt (1:200), mTOR (1:2000), phospho-mTOR (1:1000), phospho-GSK3β (1:1000), phospho-PDK1 (1:200), phospho-PTEN (1:1000), caspase 3 (1:1000), and HSP70 (1:1000). All primary antibodies were purchased from Cell Signaling Technology (USA). For detection, a secondary antibody—Goat-anti-rabbit IgG(H + L), HRP conjugate (R-05072-500, Advansta, USA)—was used at dilutions of 1:2000 or 1:1000, depending on experimental conditions. After incubation, the membrane was washed 4 times for 10 min with PBST-0.1%/TBST-0.1% solution and incubated with horseradish peroxidase–conjugated secondary anti-mouse/anti-rabbit/anti-goat antibodies for 1 h at RT. After washing, chemiluminescent signal was detected using the WesternBright ECL HRP substrate (Advansta, USA) and the Chemi-Doc XRS+ system with Image Lab software (Bio-Rad Laboratories, USA). Oversaturation of protein spots has been addressed in previous studies proposing effective detection and reconstruction techniques.^18^^,^^19^ Subsequent calculations of the blots were performed using ImageJ software (NIH, USA).

### Animal study

All animal procedures were approved by the State Food and Veterinary Service of the Republic of Lithuania (license/approval No. G2-119). Female immunodeficient mice, NOD.CB-17-Prkdc scid/Rj, were acquired from JANVIER LABS (France). The mice were 6 weeks old and weighed around 18 g, with a tolerance of ±0.5 g. They were allocated a minimum of one week to adjust to their new environment. The mice were kept in a pathogen-free environment.

To establish a chemotherapy-induced female mouse model, mice were administered a single intraperitoneal injection of cyclophosphamide at 120 mg/kg and busulfan at 30 mg/kg. After 7 days, affected female mice were categorized into 3 groups: (1) the chemotherapy-induced female mice model group (POF mice group, *n* = 6); (2) the model group of chemotherapy-induced female mice after the transplantation of hEndSCs into the ovary (POF-hEndSCs mice group, *n* = 6); (3) the model group of chemotherapy-induced female mice model group following hFFSC transplantation into the ovary (POF-hFFSCs mice group, *n* = 6); (4) the model group of chemotherapy-induced female mice after hMenSC transplantation into the ovary (POF-hMenSCs mice group, *n* = 6); and (5) the chemotherapy-induced female mice model group following hPSC transplantation into the ovary (POF-hPSCs mice group, *n* = 6). Furthermore, we had a control group of untreated mice (*n* = 6). After 7 days, all groups were segregated into 2 categories: mice that were sacrificed for further molecular study (*n* = 3 from each group) and mice that cohabitated with male mice (*n* = 3 from each group) for a fertility test. A fertility assessment was completed after 14 days. The quantity of embryo implantation sites in the uterine horns was visually inspected and recorded after dissection of the uterine horns. At the end of the experiment, ovaries and uterine horns were collected for further protein and gene expression analysis.

To evaluate AMH levels in mice serum, blood was taken and centrifuged at 2500 *g* for 10 min. The serum was collected and tested using an ELISA kit (cat.no.CK-E90200, Hangzhou East Biopharma, Ltd., China), according to the manufacturer’s protocol.

To evaluate TNF-α levels in mice serum, blood was taken and centrifuged at 2500 *g* for 10 min. The serum was collected and tested using a Mouse TNF-α High-Sensitivity ELISA Kit (Thermo Fisher Scientific, USA) according to the manufacturer’s protocol.

### Statistical analysis

Statistical analysis of the data obtained during this work was performed using the “R” software (version 4.2.1), “rstatix” (version 0.7.1), and “stats” packages. The Kruskal–Wallis criterion was selected to determine the statistical significance of the differences in quantitative data between several groups. Post-hoc analysis was performed using the Dunn test. The Fisher exact criterion was used to determine the statistical significance of the qualitative data (pregnancy rate). The significance level (α) selected for the statistical tests was 0.05.

## Results

**Figure 1.**
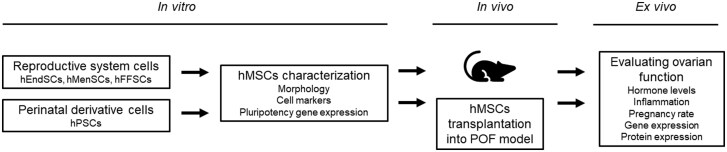
Experimental design scheme. Human mesenchymal stromal cells (hMSCs), endometrial-derived stromal cells (hEndSCs), follicular fluid stromal cells (hFFSCs), menstrual blood–derived stromal cells (hMenSCs), and placenta-derived (hPSCs) stromal cells were isolated, characterized, and then transplanted into the ovary of the POF mice model for molecular study.

We conducted a head-to-head comparison of human mesenchymal stromal cells (hMSCs) isolated from 4 reproductive/perinatal sources—endometrium (hEndSCs), menstrual blood (hMenSCs), placenta (hPSCs), and follicular fluid (hFFSCs)—to determine whether tissue of origin influences the capacity to restore reproductive function in vivo. Using a standardized chemotherapy-induced POF model, each MSC product was delivered as a single intraovarian dose at an identical time point and dose, enabling a controlled assessment of source-dependent efficacy (Figure 1).

Our primary endpoint was functional fertility (pregnancy rate; embryo counts), chosen to distinguish true reproductive rescue from partial endocrine recovery. Secondary readouts captured coordinated ovarian–uterine repair: serum AMH, ovarian folliculogenesis markers and signaling proteins (eg, PI3K/AKT/mTOR), and uterine programs of extracellular-matrix remodeling and proliferation. This design tests the a priori hypothesis that MSCs from different reproductive tissues possess distinct paracrine profiles that differentially couple follicular activation with endometrial remodeling, thereby producing source-specific therapeutic effects (Figure 1).

### Characterization of human endometrium, menstrual blood, placenta, and follicular fluid–derived stromal cells

**Figure 2.**
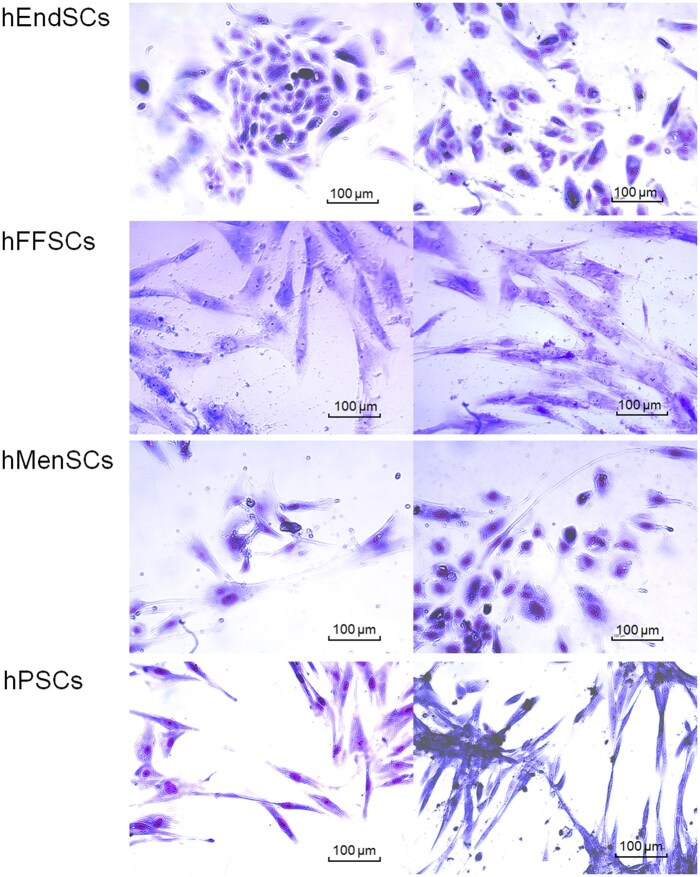
Morphology of human endometrial–derived stromal cells (hEndSCs), follicular fluid stromal cells (hFFSCs), menstrual blood–derived stromal cells (hMenSCs), and placenta-derived (hPSCs) stromal cells. Cells were stained with a Giemsa and images were captured using an inverted microscope (Motic AE2000). Scale bars are shown.

The morphology of hMSCs isolated from human tissues was characterized by Giemsa staining and light microscopy imaging (Figure 2). The hMSCs morphology was observed to be heterogeneous. hEndSCs and hMenSCs appeared to be rounder and smaller, compared to hPSCs and hFFSCs, exhibiting epithelial-like cell morphology. The hPSCs and hFFSCs were elongated and spindle-shaped, exhibiting fibroblast-like cell morphology.

**Figure 3.**
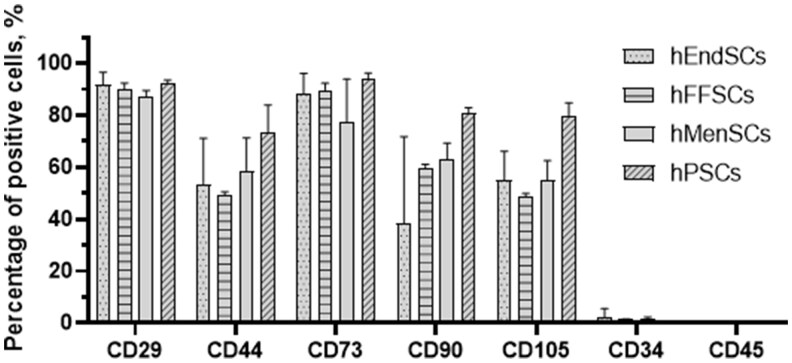
Expression of cell surface markers of human endometrial–derived stromal cells (hEndSCs), follicular fluid–derived stromal cells (hFFSCs), menstrual blood–derived stromal cells (hMenSCs), and placenta-derived (hPSCs) stromal cells. The results are presented as mean ± SD (*n* = 10). Kruskal–Wallis test: all *P* > .05.

In this study, we determined the expression of hMSCs’ surface markers CD29, CD34, CD44, CD45, CD73, CD90, and CD105 (Figure 3). hEndSCs, hMenSCs, hPSCs, and hFFSCs were positive for specific surface antigens such as CD29 (88.3%–92.4% of the total population), CD44 (48.3%–73.4% of the total population), CD73 (77.3%–93.7% of the total population), CD90 (38.5%–80.9% of the total population), and CD105 (47.7%–79.5% of the total population). We found that these cells did not express the hematopoietic lineage markers CD34 (0.1%–2.2% of the total population) and CD45 (0% of the total population). The Kruskal–Wallis test did not reveal statistically significant differences between hMSCs derived from different human tissues (CD29 *P *= .4, CD44 *P *= .4, CD73 *P *= .2, CD90 *P *= .2, CD105 *P *= .2, CD34 *P *= .4, CD45 *P* = NA). However, hFFSCs possess the highest stemness potential.

**Figure 4.**
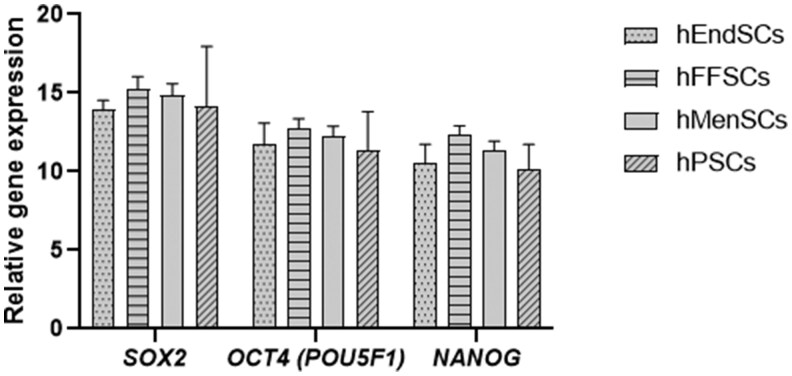
Expression of pluripotency transcription factors *SOX2*, *OCT4 (POU5F)*, and *NANOG* in human endometrial–derived stromal cells (hEndSCs), follicular fluid cells (hFFSCs), menstrual blood–derived stromal cells (hMenSCs), and placenta-derived (hPSCs) stromal cells (hPSCs). The results are presented as mean ± SD (*n* = 10). Kruskal–Wallis test: all *P* > .05.

Next, we investigated the expression of transcription factor coding genes *SOX2*, *OCT4 (POU5F)*, and *NANOG* in hEndSCs, hFFSCs, hMenSCs, and hPSCs (Figure 4). The levels of *SOX2* expression were similar across different hMSCs (hEndSCs 14 ± 0.6, hFFSCs 15.3 ± 0.8, hMenSCs 14.9 ± 0.7, hPSCs 14.2 ± 3.8). The expression levels of *OCT4 (POU5F)* in hMSCs were slightly higher than the *SOX2* levels (hEndSCs 11.8 ± 1.3, hFFSCs 12.7 ± 0.6, hMenSCs 12.3 ± 0.6, hPSCs 11.3 ± 2.5). The highest relative expression among the 3 pluripotency genes was observed for *NANOG* gene in hMSCs derived from different tissues (hEndSCs 10.6 ± 1.2, hFFSCs 12.3 ± 0.6, hMenSCs 11.4 ± 0.6, hPSCs 10.2 ± 1.6). The statistical analysis did not show significant differences between hMSCs derived from different tissue sources (*SOX2 P *= 0.6, *OCT4 [POU5F] P *= 0.8, *NANOG P *= 0.5).

### Effect of stromal cells derived from endometrium, menstrual blood, placenta, and follicular fluid on fertility restoration in POF mice model

After the assessment of hMSC morphology, surface, and pluripotency marker expression, in vivo experiments were further performed. Premature ovarian failure (POF) was induced in mice, and endometrial-derived stromal cells (hEndSCs), follicular fluid stromal cells (hFFSCs), menstrual blood–derived stromal cells (hMenSCs), and placenta-derived stromal cells (hPSCs) transplantation into the mouse ovary was performed. After the procedures, parameters related to mouse fertility and ovarian function, AMH, fertility efficiency, and expression of genes and proteins related to folliculogenesis or regeneration processes were evaluated.

**Figure 5.**
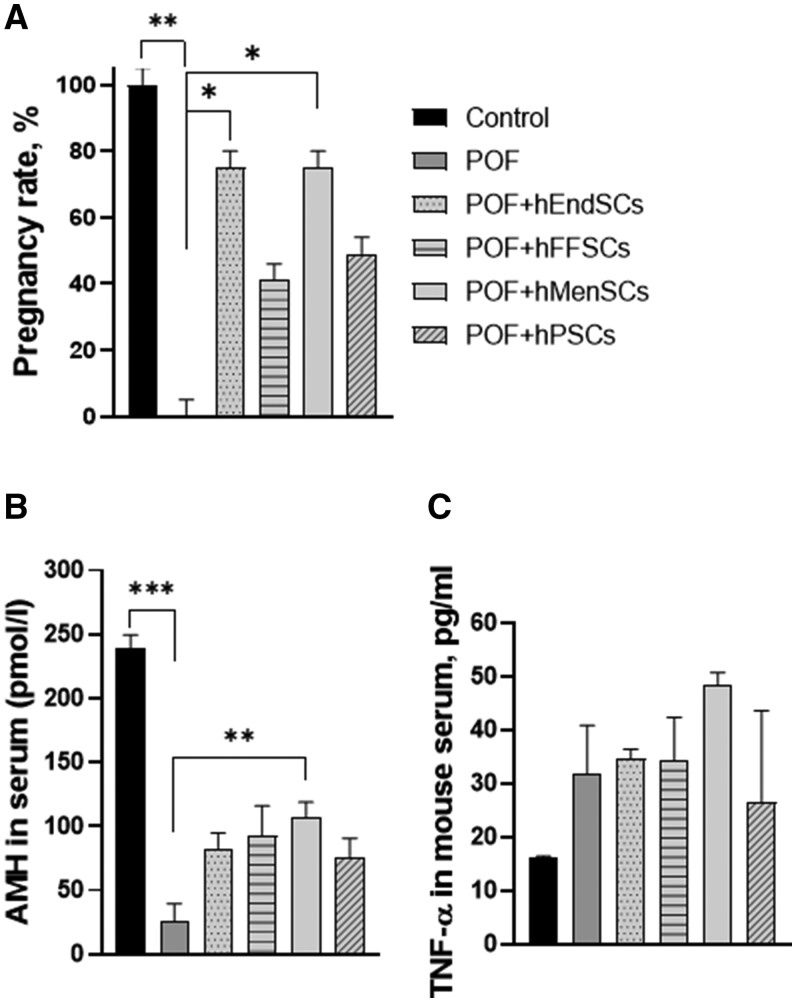
POF mouse fertility evaluation after hMSCs treatment. The mouse pregnancy rate (A), AMH concentration in mouse serum (B), and TNF-α concentration in mouse serum (C) were evaluated. Control (untreated), POF (premature ovarian failure), POF model treated with endometrial (POF + hEndSCs), menstrual blood (POF + hMenSCs), placenta (POF + hPSCs), and follicular fluid (POF + hFFSCs)–derived MSCs. Group size: *n* = 6 females per group; fertility assay in panel A used *n* = 3 mated females per group; serum assays in panels B and C used *n* = 3 females per group. Data are presented as mean ± SD. Statistical significance is denoted by asterisks (*P < .05, **P < .01, ***P < .001).

The efficacy of hMSCs in treating POF was first determined by observing the mouse pregnancy rate 2 weeks after the procedures (Figure 5A). Treatment groups were compared using Fisher’s exact test with the control and POF groups. The mice in the control group became pregnant within a week of mating and the pregnancy rate was 100% (litter size ranged from 6 to 12 pups). The results of the POF group showed an established POF model with a pregnancy rate of 0% (experiment was carried out for 2 months). The differences between the pregnancy rates of the control and POF groups were statistically significant (*P *= .008). Treatment with hMSCs from different sources revealed various levels of treatment efficiency. POF mouse treatment with hEndSCs and hMenSCs was the most effective at restoring mouse fertility (pregnancy rate 75%), and the result was statistically significant when compared with POF group (*P *= .048 for both treatment groups) and not significant when compared with the control group (*P *= 1). Treatment with hPSCs increased the pregnancy rate up to 49% (compared to the control group, and the POF group, *P *= .1) and hFFSCs treatment increased the pregnancy rate up to 41% (compared with control group *P *= .04, and with POF group *P *= .2).

Anti-Müllerian hormone (AMH) levels in mouse serum were used as a biomarker of ovarian function. The multiple comparison test revealed statistically significant differences between different treatment groups (*P *< .001). The control group had the highest concentration of AMH in mouse serum (239 pmol/L), and the POF group had the lowest (25 pmol/L), revealing disruption of ovarian function (Figure 5B), and the difference between these groups was statistically significant (*P *< .001). POF + hMenSCs and POF + hFFSCs treatments showed the greatest effectiveness in restoring AMH concentration (107 pmol/L and 92 pmol/L, respectively). POF + hMenSCs group’s AMH results were significantly different from the results of the POF group (*P *= .002); however, POF + hFFSCs results were not significant (*P *= .06). POF + hPSCs and POF + hEndSCs treatments restored twice the AMH levels compared with the POF model (75 pmol/L and 83 pmol/L, respectively); however, the difference was not significant (POF + hPSCs *P *= .06, POF + hEndSCs *P* = .06).

We evaluated the anti-inflammatory effects of hMSCs by measuring the levels of inflammatory factor TNF-α in mouse serum with ELISA (Figure 5C). TNF-α concentration in serum almost doubled 1–2 days after POF model was established by chemotherapy (31.7 pg/mL) when compared with the control group (16.2 pg/mL). POF mouse treatment with hMSCs did not lower the levels of TNF-α, and the concentration was comparable to the POF group 1–2 days after chemotherapy treatment (hEndSCs 34.7 pg/mL, hFFSCs 34.2 pg/mL, hMenSCs 48.3 pg/mL, hPSCs 26.5 pg/mL). Statistical analysis with the Kruskal–Wallis test did not show statistically significant differences between the experimental groups (*P *= .08).

### Changes in gene and protein expression in mouse ovary tissue after stromal cell treatment

To evaluate the molecular effects of stromal cell therapy on the premature ovarian failure (POF) mice model, POF was induced in mice, and after a week, single-dose cell treatments of human endometrial (hEndSC), follicular fluid (hFFSC), menstrual blood (hMenSC), or placental (hPSC) stromal cells were administered. The RT-qPCR technique was used to analyze the changes in the expression of folliculogenesis-associated genes (*Amh, Ctgf, Fshr, Gdf3, Gja1, Inha, Mvh,* and *Zp1*) in mouse ovary tissue and Western blot analysis to assess protein expression levels in mouse ovary tissue.

**Figure 6.**
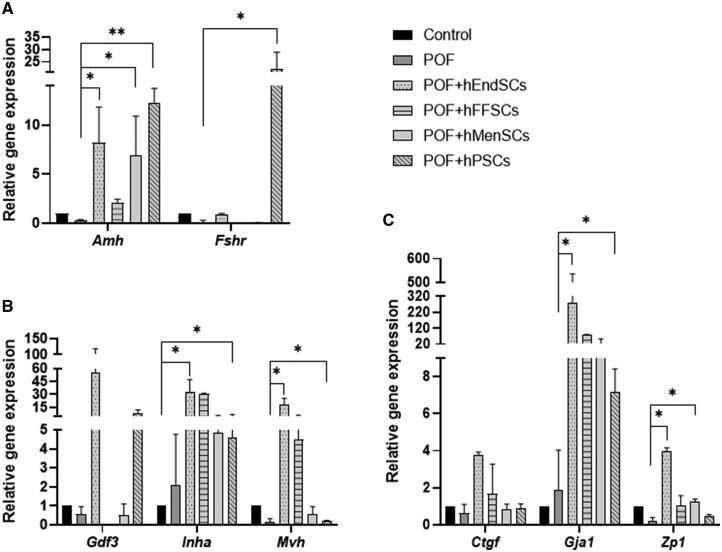
Gene expression profiles of mouse ovary tissue samples of premature ovarian failure (POF) model and after stromal cell therapy treatment. The expression of (A) the *Amh* and *Fshr* genes; (B) the *Gdf3*, *Inha*, and *Mvh* genes, and (C) *Ctgf*, *Gja1*, and *Zp1* genes was evaluated by RT-qPCR. Control (untreated), POF (premature ovarian failure), POF model treated with endometrial (POF + hEndSCs), menstrual blood (POF + hMenSCs), placenta (POF + hPSCs), and follicular fluid (POF + hFFSCs) derived MSCs. mRNA expression levels were normalized to the geometric mean of *Actb* and *18 s,* and relative gene expression was calculated using ΔΔCt method. Results are presented as mean ± SD (*n* = 3). Statistical analysis was performed using the Kruskal–Wallis test, where *P ≤ .05; **P ≤ .01.

We investigated the expression of *Amh*, *Ctgf*, *Fshr*, *Gdf3*, *Gja1*, *Inha*, *Mvh*, and *Zp1* genes in mouse ovary tissue. Multiple comparisons with the Kruskal–Wallis test revealed significant differences in the treatment groups for the *Amh*, *Fshr*, *Gdf3*, *Gja1*, *Inha*, *Mvh*, and *Zp1* genes (*P *= .002, *P *= .01, *P *= .02, *P *= .009, *P *= .03, *P *= .008, *P *= .008, respectively), while the expression of the *Ctgf* gene in the treatment groups did not change significantly (*P *= .54, *P *= .18, respectively). *Post-hoc* analyses were performed for *Amh*, *Fshr*, *Gdf3*, *Gja1*, *Inha*, *Mvh*, and *Zp1* genes. We compared the gene expression between control and POF mouse ovary tissues; the genes *Amh* (0.3 ± 0.1 fold change [FC], *P *= .29), *Ctgf* (0.6 ± 0.5 FC), *Fshr* (0.1 ± 0.2 FC, *P *= .07), *Gdf3* (0.6 ± 0.4 FC, *p *= 0.3), *Mvh* (0.2 ± 0.2 0 FC, *p *= 0.07), and *Zp1* (0.2 ± 0.2 FC, *P *= .1) were downregulated and the genes *Gja1* (1.9 ± 2.1 FC, *p *= 0.2) and *Inha* (2.1 ± 2.7 FC, *P *= .2) were upregulated; however, the differences were statistically insignificant. The expression of the *Amh* gene was significantly restored in POF + hEndSCs (8.2 ± 3.6 FC, *P *= .03), POF + hMenSCs (6.94 ± 4 FC, *P *= .01), POF + hPSCs (12.2 ± 0.4 FC, *P *= .008) groups compared to the POF group. Treatment with hFFSCs also upregulated *Amh* gene expression (2.1 ± 0.4 FC, *P *= .2). The expression of the *Fshr* gene was recovered only in POF + hPSCs treatment group (22.1 ± 6.8 FC, *P *= .01), while in POF + hEndSCs (0.9 ± 0.07 FC, *P *= .1), POF + hMenSCs (0.05 ± 0.05 FC, *P *= .4), POF + hFFSCs (0.04 ± 0.04 FC, *P *= .4) groups remained downregulated (Figure 6A). *Gdf3* mRNA levels in POF mouse ovary tissue were restored by treatment with hEndSCs (55.9 ± 62.8 FC, *P *= .07) and hFFSCs (7.7 ± 4.6 FC, *P *= .1), but not with hMenSCs (0.5 ± 0.6 FC, *P *= .4) and hPSCs (0.04 ± 0.001 FC, *P *= .09). In all treatment groups, *Inha* mRNA levels remained elevated (POF + hEndSCs [32.3 ± 14.8 FC, *P *= .07], POF + hFFSCs [4.6 ± 2.2 FC, *P *= .5], POF + hMenSCs [4.8 ± 1 FC, *P *= .4], POF + hPSCs [4.5 ± 1.6 FC, *P *= .09]). The expression of the *Mvh* gene was restored in POF + hEndSCs (18.5 ± 7 FC,*P *= .02) and POF + hPSCs (4.5 ± 1.6 FC, *P *= .04), and remained downregulated in POF + hMenSCs (0.6 ± 0.4 FC, *P *= .1) and POF + hFFSCs (0.2 ± 0.03 FC, *P *= .3) (Figure 6B). *Ctgf* mRNA levels were restored in POF + hEndSCs (3.8 ± 0.2 FC) and POF + hPSCs (1.7 ± 1.6 FC treatment groups, while in POF + hMenSCs (0.8 ± 0.3 FC) and POF + hFFSCs (0.9 ± 0.2 FC) remained downregulated. *Gja1* gene expression was restored by all hMSC treatments; in POF + hEndSCs (278.4 ± 257.7 FC, *P *= .02) and POF + hPSCs (78.5 ± 1.2 FC, *P *= .03) treatment groups, the differences were statistically significant; however, in POF + hFFSCs (7.2 ± 1.3 FC, *P *= .2) and POF + hMenSCs (23.4 ± 30 FC, *P *= .2) groups, differences were not significant. *Zp1* mRNA levels were restored after treatment with POF + hEndSCs (4 ± 0.2 FC, *P *= .01) and POF + hMenSCs (1.2 ± 0.2 FC, *P *= .02), but not in POF + hPSCs (1 ± 0.6 FC, *P *= .1) and POF + hFFSCs groups (0.5 ± 0.07 FC, *P *= .3) (Figure 6C).

**Figure 7.**
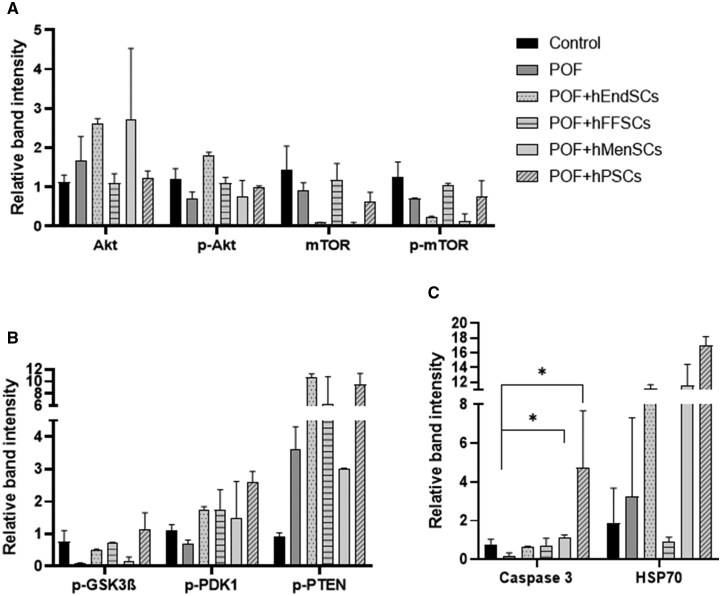
Protein expression profiles of mouse ovarian tissue samples from the premature ovarian failure (POF) model and after stromal cell therapy treatment. Expression of (A) Akt, phosphorylated Akt (p-Akt), mTOR, phosphorylated mTOR (p-mTOR), (B) phosphorylated GSK3β (p-GSK3β), phosphorylated PDK1 (pPDK1), phosphorylated PTEN (p-PTEN), and (C) Caspase 3, HSP70 was evaluated by Western blot. Control (untreated), POF (premature ovarian failure), POF model treated with endometrial (POF + hEndSCs), menstrual blood (POF + hMenSCs), placenta (POF + hPSCs), and follicular fluid (POF + hFFSCs)–derived MSCs. The results are presented as mean ± SD, *n* = 3. Statistical significance is denoted by asterisks (*P < .05). Full-size, uncropped blots are provided in Figure S1 (see online supplementary material for a color version of this figure).

Next, we performed Western blot quantification to evaluate changes in folliculogenesis-associated protein quantity after POF mouse treatment with hMSCs. The results of Akt and phosphorylated Akt (p-Akt) differed from each other (Figure 7A, representative bands of detected proteins are presented in Figure S1, see online supplementary material for a color version of this figure). Akt protein levels were upregulated after chemotherapy (1.7 ± 0.6), in POF + hEndSCs (2.6 ± 0.1), and in POF + hMenSCs (2.7 ± 1.8) treatment groups. In POF + hFFSCs (1.1 ± 0.2) and POF + hPSCs (1.2 ± 0.2) treatment groups, Akt levels were comparable to the control group (1.1 ± 0.2). Akt activated form p-Akt was downregulated in POF group (0.7 ± 0.2) and in POF + hMenSCs group (0.8 ± 0.4). p-Akt levels were restored to the levels the control group (1.2 ± 0.3) in POF + hEndSCs (1.8 ± 0.09), POF + hFFSCs (1.1 ± 0.1), and POF + hPSCs (1.0 ± 0.03) groups. For both Akt and p-Akt multiple comparisons, the statistical test revealed no significant differences between the groups (Akt *P *= .3, p-Akt *P *= .3). mTOR and phosphorylated mTOR (p-mTOR) levels were lowered in POF model (0.9 ± 0.2 and 0.7 ± 0.02 respectively) and restored in POF + hFFSCs group (1.2 ± 0.4 and 1.1 ± 0.04, respectively) to the levels of the control group (1.4 ± 0.6 and 1.3 ± 0.4, respectively) (Figure 7A). A decrease in mTOR and p-mTOR protein expression was observed in POF + hEndSCs (0.1 ± 0.01 and 0.2 ± 0.01, respectively), POF + hMenSCs (0.04 ± 0.06 and 0.1 ± 0.2, respectively), and POF + hPSCs (0.6 ± 0.2, 0.8 ± 0.4, respectively) treatment groups. However, the differences between groups were not statistically significant (mTOR *P *= .1, p-mTOR *P *= .2). Phosphorylated GSK3β (p-GSK3β) protein was downregulated in POF (0.1 ± 0.02), POF + hEndSCs (0.5 ± 0.03), and POF + hMenSCs (0.1 ± 0.1) groups. p-GSK3β protein levels were restored in POF + hFFSC (0.7 ± 0.03) and POF + hPSCs (1.2 ± 0.5) groups to the level of the control group (0.8 ± 0.3). The phosphorylated protein PDK-1 (p-PDK-1) was downregulated in POF model (0.7 ± 0.1) and positively regulated in groups POF + hEndSCs (1.8 ± 0.09), POF + hMenSCs (1.5 ± 1.1), POF + hFFSCs (1.7 ± 0.6), and POF + hPSCs (2.6 ± 0.3) when comparing with the control group (1.1 ± 0.2). Phosphorylated PTEN (p-PTEN) levels were upregulated in POF (3.6 ± 0.7), POF + hEndSCs (10.7 ± 0.5), POF + hMenSCs (3 ± 0.03), POF + hFFSCs (6.2 ± 4.6), and POF + hPSCs (9.5 ± 1.8) groups when compared with the control group (0.9 ± 0.1) (Figure 7B). Different treatment groups did not reveal significant differences for p-GSK3β (*P *= .1), p-PDK-1 (*P *= .2), and p-PTEN (*P *= .1) proteins. For Caspase 3, the multiple comparison test showed significant differences between groups (*P *= .03) and post hoc analysis was performed. Caspase 3 protein expression was reduced in POF group (0.2 ± 0.2) comparing with the control group (0.7 ± 0.3) (*P *= .2). After POF + hEndSCs (0.7 ± 0.03), POF + hMenSCs (1.1 ± 0.1), POF + hFFSCs (0.7 ± 0.3), and POF + hPSCs (4.7 ± 2.9) treatments, caspase 3 expression was restored to the control level. Post hoc analysis revealed significant differences between POF and POF + hMenSCs groups (*P *= .03) and between POF and POF + hPSCs groups (*P *= .01). The comparison between POF and POF + hEndSCs (*P *= .3) and POF + hFFSCs (*P *= .2) revealed that the differences were not significant. HSP70 was upregulated in POF (3.2 ± 4) compared to the control group (1.8 ± 1.8). In the treatment groups, HSP70 was downregulated in POF + hFFSCs (0.9 ± 0.2) and upregulated in POF + hEndSCs (11.1 ± 0.6), POF + hMenSCs (11.5 ± 3), and POF + hPSCs (17 ± 1) (Figure 7C). However, the results of HSP70 expression in mouse ovaries were not statistically significant (*P *= .06).

### Changes in gene and protein expression in mouse uterus tissue after stromal cell treatment

To evaluate the changes in gene and protein levels in uterus tissue of the POF mice model, the RT-qPCR technique was used. We analyzed the endometrial markers (*Pdgfr, Mcam, Foxa2, Cxcl15*), markers of wound healing and regeneration (*Col1a1, Col3a1, Ctgf*), and proliferation and cell cycle progression markers (*Pcna, Ccnd1, Ki67*) after stromal cells derived from human endometrium (hEndSC), follicular fluid (hFFSC), menstrual blood (hMenSC), or placenta (hPSC) were transplanted into POF mice ovaries.

**Figure 8.**
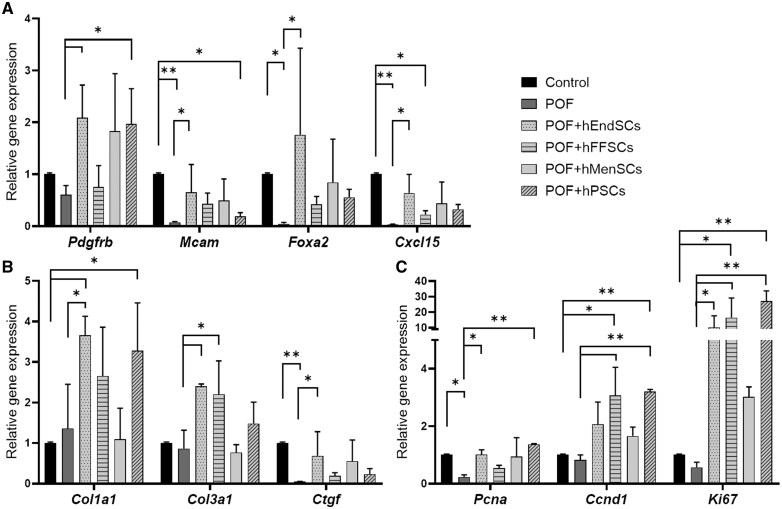
Gene expression profiles of mouse uterine tissue samples from the POF model and after treatment with stromal cell therapy. The expression of (A) genes associated with endometrial function *Pdgfr*, *Mcam*, *Foxa2*, and *Cxcl15*; (B) genes related to wound healing and regeneration *Col1a1*, *Col3a1*, and *Ctgf*; and (C) genes involved in proliferation and cell cycle progression *Pcna*, *Ccnd1*, and *Ki67* was analyzed using RT-qPCR. Control (untreated), POF (premature ovarian failure), POF model treated with endometrial (POF + hEndSCs), menstrual blood (POF + hMenSCs), placenta (POF + hPSCs), and follicular fluid (POF + hFFSCs)–derived MSCs. mRNA expression levels were normalized to the geometric mean of *Actb* and *18 s*, and relative gene expression was calculated using ΔΔCt method. Results are presented as mean ± SD (*n* = 3). Statistical analysis was performed using the Kruskal–Wallis test, where *P ≤ .05; **P ≤ .01.

Results revealed that in POF mice model, the expression of almost all analyzed genes was downregulated, and compared to control mice, significant changes were detected in *Mcam*, *Foxa2*, *Cxcl15*, *Cyp19a*, *Ctgf*, and *Pcna* gene expression (approx. 14-fold, 22-fold, 33-fold, 19-fold, 19-fold, and 4-fold, respectively) (Figure 8). We also determined that the expression of genes related to endometrial function and health was increased after stromal cell treatments when compared to the POF model, and the most effective cell source was proven to be hEndSCs, since the upregulation of all genes tested was noted to be significant (Figure 8A). Furthermore, the expression of *Pdgfrb* also increased significantly after treatment with hPSCs. Also, the gene expression of proteins associated with wound healing was also analyzed, and it was noted that using hEndSC treatments resulted in significant expression changes of *Col1a1, Col3a1*, and *Ctgf* genes, compared to the POF group (Figure 8B). A significant change in Col3a1 expression was observed after treatment with hFFSCs. To track cell proliferation and renewal, gene expression of cell cycle proteins was examined, and noticeable and significant expression upregulation when compared to the POF model was observed using hEndSCs (*Pcna* 4.4-fold, *Ki67* 17.5-fold increase), hFFSCs (*Ccnd1* 3.7-fold, *Ki67* 28.8-fold increase), and hPSCs (*Pcna* 5.9-fold, Ccnd1 3.8-fold, *Ki67* 47-fold increase) (Figure 8C).

After treatments with stromal cells derived from various sources of the reproductive system and perinatal derivatives were administered to the POF mice model, the treatment with hEndSCs proved to be the most prolific, since significant gene expression upregulation was determined in most of the tested genes. We also found that hFFSCs and hPSCs treatments affected the expression profile of the tested genes, especially those related to the cell cycle.

**Figure 9.**
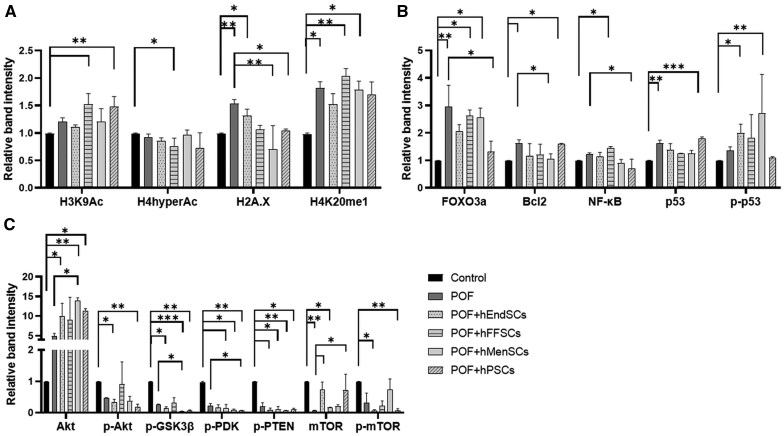
Protein expression profiles of mouse uterine tissue samples from the premature ovarian failure (POF) model and after treatment with stromal cell therapy. Proteins associated with (A) epigenetic regulation, (B) cell cycle regulation, and (C) endometrial function were analyzed using Western blot analysis. Results are presented as mean and ± SD (*n* = 3). Statistical analysis was calculated using the Kruskal–Wallis test, where *P ≤ .05; **P ≤ .01; ***P ≤ .001. Full-size, uncropped blots are provided in Figure S2 (see online supplementary material for a color version of this figure).

To further analyze the effect of hMSC treatment on molecular changes in uterus of POF mice model, we investigated proteins associated with epigenetic modification, cell cycle regulation, and endometrial function (Figure 9). Our results show that in most cases, proteins associated with epigenetic regulation tend to increase their level in the POF model compared to the control and remain at a similar level after stromal cell treatments (Figure 9A, representative bands of detected proteins are presented in Figure S2, see online supplementary material for a color version of this figure). An exception is H2A.X protein, where the level of this protein has increased significantly after POF was induced compared to the untreated control, but decreased after mice were injected with hMenSCs and hPSCs (Figure 9A). Furthermore, the level of modified histone associated with transcriptionally active chromatin H4K20me1 increased significantly in the POF group, but remained at a similar level after stromal cell treatment. The effects of stromal cell therapy after induced POF were further observed by analyzing cell cycle regulators (Figure 9B). However, it was revealed that the levels of the FOXO3a and Bcl2 increased significantly in the POF group, but decreased in response to the treatment with hPSCs and hMenSCs, respectively (Figure 9B). Significantly lower levels were observed in nuclear factor NF-κB after treatment with hMenSCs when compared to POF model (Figure 9B). Moreover, tumor suppressor protein p53 was upregulated in POF group in contrast to the control; however, no significant changes in protein production were observed after treatments with stromal cells.

The last group of proteins we analyzed was associated with endometrial functionality (Figure 9C). As seen in the results, most of these proteins tend to downregulate their production when phosphorylated. After using hMenSCs and hPSCs for treatments, significant downregulation of protein levels was observed in p-GSK3β and p-PDK proteins, respectively (Figure 9C). On the contrary, the investigation revealed that mTOR increased its level after treatment with hEndSCs and hPSCs and Akt increased its production after treatment with hMenSCs compared to POF group.

Overall, the most significant changes in protein production can be seen after therapy with hMenSCs and hPSCs stromal cells.

## Discussion

This study provides a source-resolved view of mesenchymal stromal cell (MSC) therapy for reproductive repair by testing 4 reproductive/perinatal MSC types—hEndSCs, hMenSCs, hPSCs, and hFFSCs—side-by-side under identical dose, route, and timing in a severe chemotherapy-induced POF model. Within 7 days of Bu/Cy exposure, we documented a pronounced reproductive damage characterized by uterine wall thinning/fibrosis and subsequent infertility in mating trials, establishing a stringent baseline for testing reparative interventions.^18^ Given that the murine estrous cycle lasts approximately 5 days, our used 7–14 days’ timeframe already encompasses 2 to 3 natural cycles, permitting the assessment of early functional recovery. All cell products met ISCT minimal criteria, minimizing identity/quality confounders and allowing efficacy to be attributed to tissue-of-origin biology.^20^ In this study, we showed that all hMSC show functional fertility rescue (41%–75% pregnancy) rather than endocrine improvement alone, with hEndSCs and hMenSCs reproducibly achieving the highest pregnancy rates (both 75%) and AMH recovery. Second, we recorded this benefit to dual-compartment mechanisms—concurrent ovarian follicular activation and uterine remodeling—rather than focusing on the ovary in isolation. Finaly, we identified source-specific molecular signatures, including a unique *Fshr* upregulation with hPSCs, that argue for rational source selection in future translational use.

At the ovarian level, MSC therapy increased *Amh* expression and serum AMH, consistent with partial restoration of the follicular pool, and differentially recovered genes supporting granulosa–oocyte communication and oocyte competence (*Gja1, Zp1, Ddx4/Mvh*).^21^^,^^22^ The increase in *Gdf3* after hEndSC and hPSC delivery aligns with enhanced follicular growth cues.^23^ Notably, *Fshr* upregulated only after hPSC treatment, suggesting placenta-derived cues can preferentially tune gonadotropin responsiveness.^24^ Across sources, protein signaling converged on PI3K/AKT/mTOR (p-AKT, p-mTOR, p-PDK1) with restoration of p-GSK3β—pathways implicated in granulosa proliferation, oocyte–granulosa crosstalk, and primordial follicle activation.^25–28^ We did not observe changes in pPTEN, indicating that upstream restraint of the pathway may persist even as downstream effectors recover.^29^ Caspase-3 and HSP70 remained elevated, pointing to ongoing turnover/stress during remodeling rather than complete resolution at the analyzed time point.

At the uterine level, MSCs engaged programs linked to extracellular matrix rebuilding and proliferative renewal, with upregulation of *Col1a1, Col3a1, Ctgf, Pcna, Ccnd1*, and *Ki67*. The expression of *Pdgfrb, Mcam, Foxa2, Cxcl15, Col1a1, Col3a1, Ctgf, Pcna, Ccnd1*, and *Ki67* genes shows active ECM remodeling and stromal–epithelial interaction and proliferative activation, which forms the basis of endometrial receptivity. The uterine glandular epithelium maintains receptivity through LIF and other paracrine factor secretion, and the implantation window is characterized by integrin-based adhesion and extracellular matrix remodeling.^30–32^ The study shows *Foxa2* and *Cxcl15* expression patterns that match LIF-induced glandular cell activation and *Mcam* and *Col1a1* and *Col3a1* and *Ctgf* expression patterns that indicate integrin-dependent ECM and adhesion structure changes. The molecular data we obtained in this study matches the proven biological structure of a receptive endometrium that regenerates while providing detailed mechanistic information beyond basic histological descriptions. Although *Ctgf* has been associated with fibrosis, expression in our study increased relative to POI but not beyond healthy controls, favoring a healing rather than scarring interpretation.^33–35^ Source-dependent effects were again evident: hEndSCs most robustly boosted endometrial function/wound-healing genes, whereas hFFSCs/hPSCs preferentially enhanced cell-cycle regulators. Together with literature showing that reproductive MSCs can reduce fibrosis and thicken damaged endometrium, these data support a model in which MSC paracrine signals coordinate matrix organization and epithelial–stromal proliferation to improve receptivity.^36^^,^^37^ The observed beneficial effects in both ovarian and uterine compartments are likely mediated by paracrine signaling rather than direct cell engraftment. Several studies have shown that reproductive-tissue–derived MSCs produce a variety of bioactive factors—including VEGF, IGF, HGF, FGF2, CXCL12, PDGF, and TGF-β—that stimulate angiogenesis, extracellular matrix (ECM) remodeling, epithelial proliferation, and stromal repair.^38–40^ Our molecular data are consistent with these pathways: increased *Col1a1*, *Col3a1*, and *Ctgf* expression reflects matrix reorganization driven by growth factor–responsive fibroblast activation, while elevated *Pcna*, *Ccnd1*, and *Ki67* demonstrates the proliferative response typical of growth factor stimulation. In the ovary, selective induction of Fshr by hPSCs suggests a specific FSH/IGF-related paracrine axis, consistent with prior reports showing placenta-derived MSCs secrete gonadotropin-modulatory cytokines.^17^ The 2 most effective sources—hEndSCs and hMenSCs—likely act through secretion of VEGF and HGF, which are known to promote revascularization and endometrial regeneration.^36^^,^^37^ Collectively, these findings support a dual-compartment paracrine mechanism whereby MSC-derived growth factors coordinate ovarian follicular activation and uterine receptivity pathways in a synchronized manner. In contrast to reports linking endometrial dysfunction to altered PI3K/AKT dynamics, we observed down-trending p-AKT/p-PTEN vs. POI and control at this early readout, consistent with a temporal re-balancing rather than global hyperactivation; longer time-course studies will be needed to resolve this kinetics.^41–45^ Finally, systemic TNF-α was unchanged, suggesting MSC effects were localized to reproductive tissues rather than driven by broad serum-level immunomodulation.^46^

In this study, we revealed that ovarian–uterine repair is achievable with a single local MSC dose, and functional pregnancy is the clearest discriminator of true rescue. Second, tissue of origin matters: endometrium and menstrual blood provided the most consistent dual-compartment regeneration, while placenta showed a distinctive gonadotropin-sensitizing signature (*Fshr*). This argues against a one-size-fits-all MSC product and toward indication-matched source selection or combined-source strategies to couple follicular activation with endometrial remodeling.

In conclusion, by comparing 4 MSC sources under identical conditions and aligning functional fertility with endocrine and tissue programs, we show that reproductive/perinatal MSCs can rescue fertility in POF and that hEndSCs/hMenSCs are leading candidates for translation. The source-specific ovarian and uterine signatures reported here provide a blueprint for rational source selection and next-generation, mechanism-guided MSC therapies for infertility. These findings suggest potential utility not only for restoring fertility but also, prospectively, for conditions linked to ovarian aging and impaired uterine receptivity; confirmation will require purpose-designed studies.

## Supplementary Material

szag013_Supplementary_Data

## Data Availability

The data that support the findings of this study are available from the corresponding author upon reasonable request.
