# Supplementary material for: Reproductive tissue–derived stromal cells rescue fertility by coupling follicular activation with endometrial remodeling
Source: Stem Cells Transl Med. 2026 Mar 27;15(4):szag013. doi: 10.1093/stcltm/szag013 (PMC13021361; doi:10.1093/stcltm/szag013)
Supplement: szag013_Supplementary_Data [file szag013_supplementary_data.docx]

**Reproductive Tissue–Derived Stromal Cells Rescue Fertility by Coupling Follicular Activation with Endometrial Remodeling.**

Running title: Stromal cells couple ovary–uterus repair

Veronika Viktorija Borutinskaitė1*, Indrė Krastinaitė1, Elvina Valatkaitė1, Aistė Zentelytė-Vilkė1, Rūta Navakauskienė1

1-Department of Molecular Cell Biology, Institute of Biochemistry, Life Sciences Center, Vilnius University, Sauletekio av. 7, LT-01257 Vilnius, Lithuania

*Corresponding author: V. V. Borutinskaitė, PhD, Department of Molecular Cell Biology, Institute of Biochemistry, Life Sciences Center, Vilnius University, Sauletekio av. 7, LT-01257, Vilnius, Lithuania, ([veronika.borutinskaite@bchi.vu.lt](mailto:veronika.borutinskaite@bchi.vu.lt)).

**Supplementary Information**


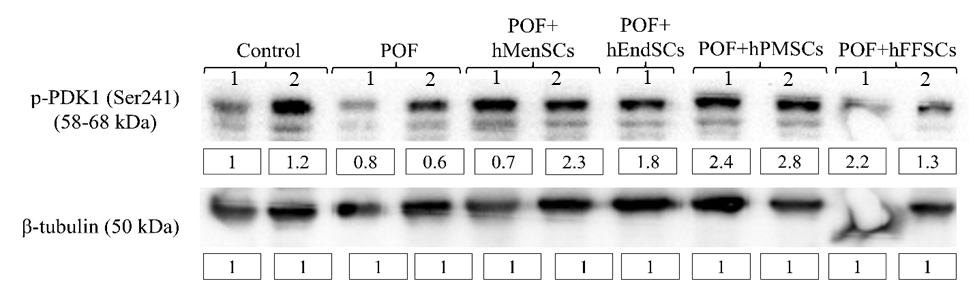


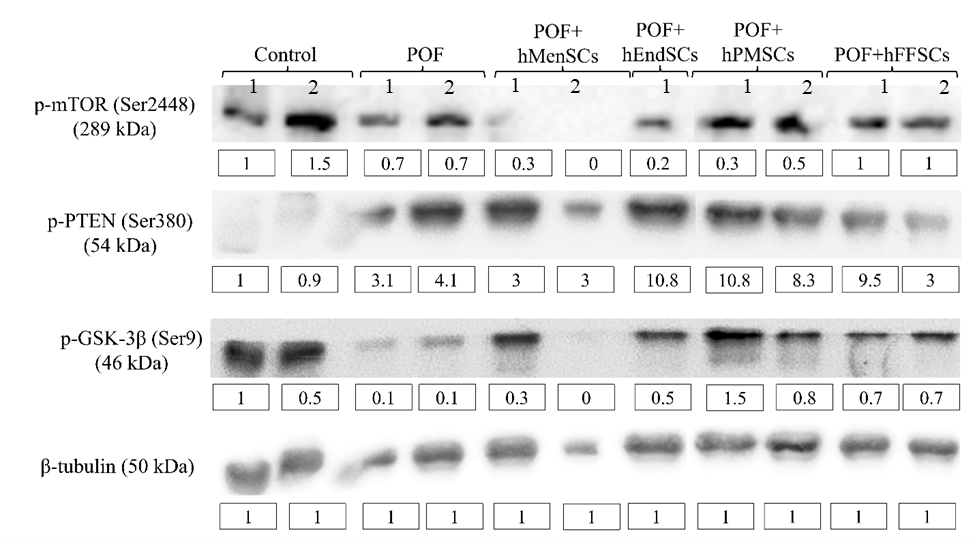


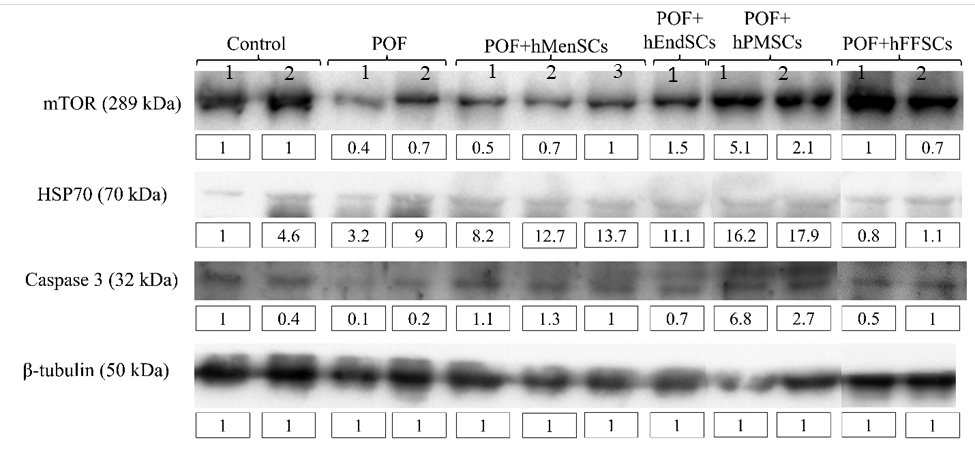


1

2

1

2

1

2

1

2

1

2

1


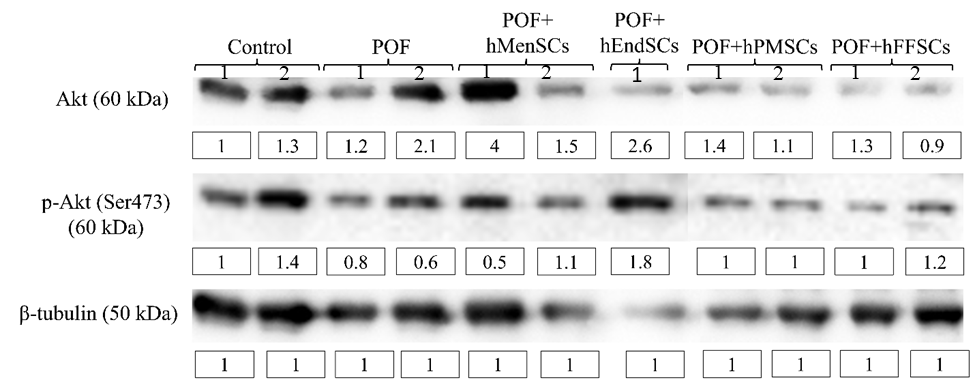


**Supplementary Figure 1.** Representative figure of protein bands representing protein levels in mouse ovarian tissues. Protein levels associated with epigenetic, cell cycle regulation and endometrial functionality were assessed after premature ovarian failure and stromal cell treatment (hEndSCs, hFFSCs, hMenSCs and hPSCs) using Western blot analysis (n=2). Relative band intensity was calculated using ImageJ software and presented in Figure 7.

Control

POF

POF+
hEndSCs

POF+
hPMSCs

POF+
hFFSCs

POF+
hMenSCs

1

1

2

1

2

1

2

1

2

1

2


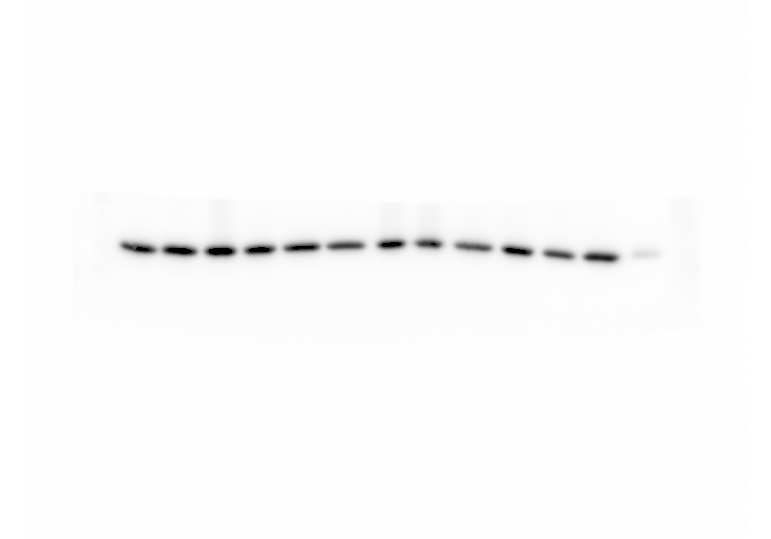

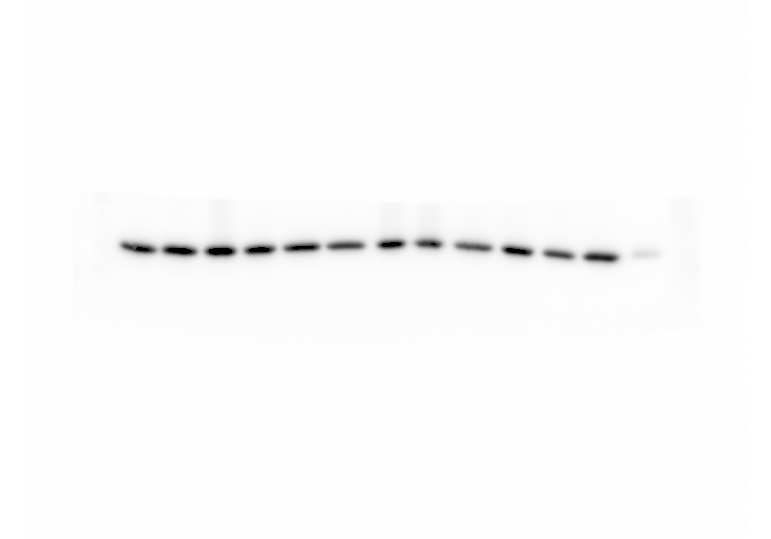


H3K9Ac (17 kDa)


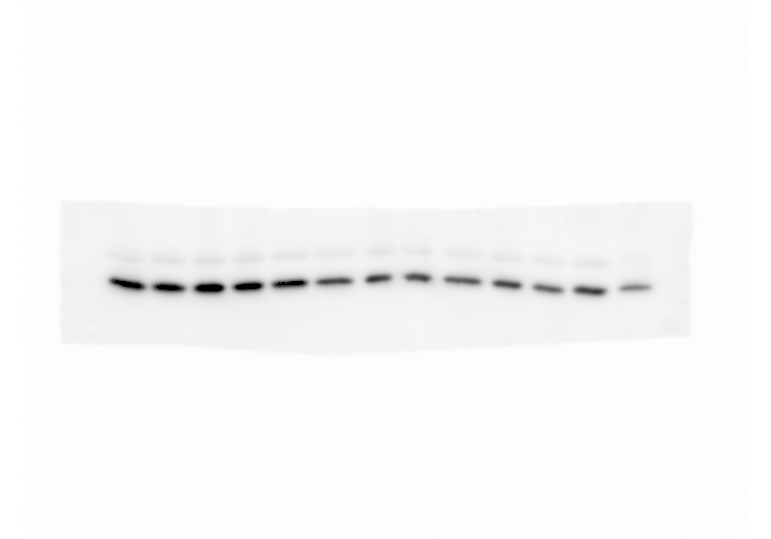

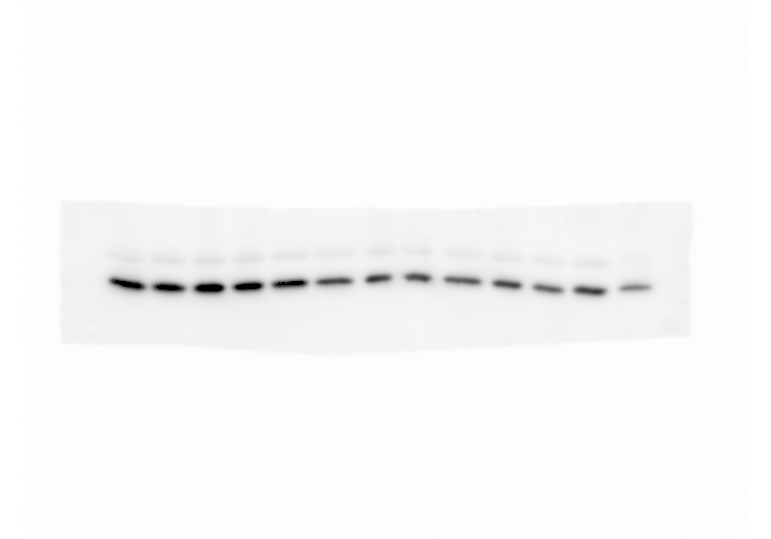


1

1

1

1

1

1

1

1

1

1

1

1

1

1.3

1.3

1.7

1.4

1

1.7

1.3

1.2

1.1

1.1

H4 (11 kDa)

Control

POF

POF+
hEndSCs

POF+
hPMSCs

POF+
hFFSCs

POF+
hMenSCs

1

1

2

1

2

1

2

1

2

1

2


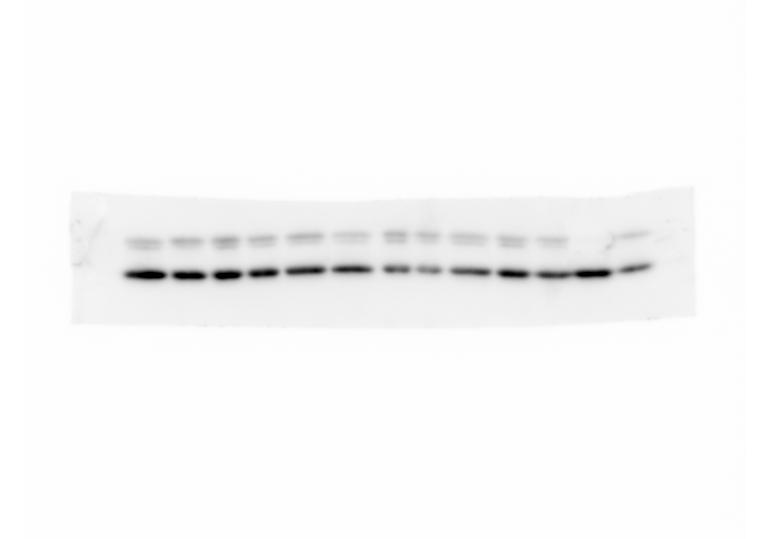

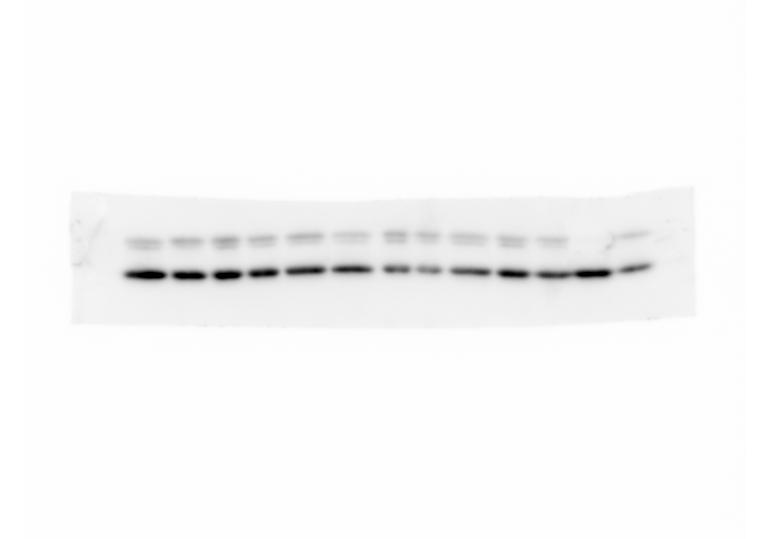


H4hyperAc (11 kDa)

1

0.9

0.8

0.9

1

0.5

1

0.6

1

1.1

0.9


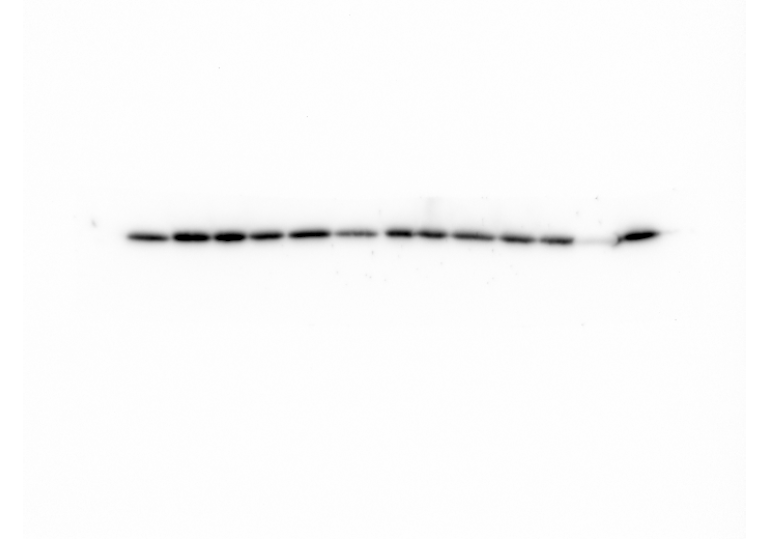

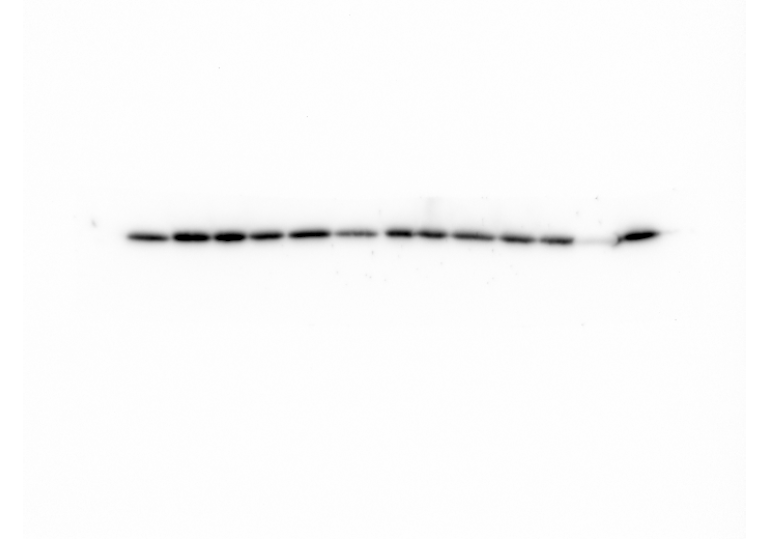


H2A.X (15 kDa)

1

1.5

1.2

1.4

1.1

1

1.6

1

1.1

0.3

1.1


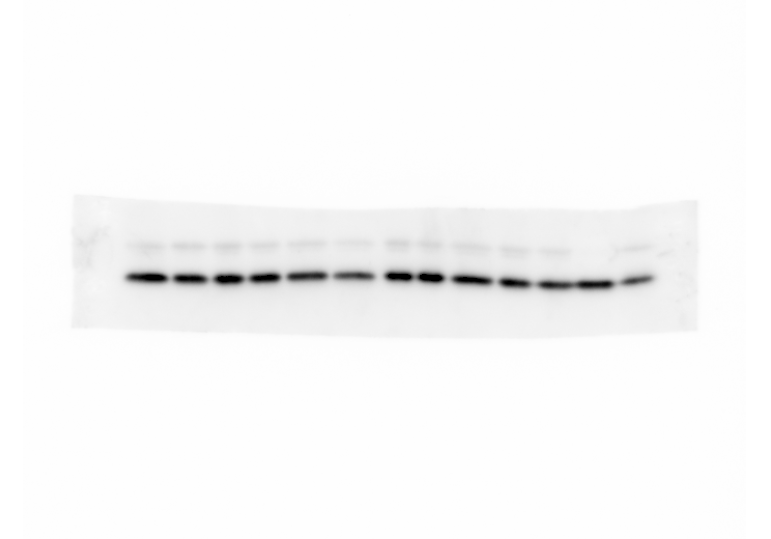

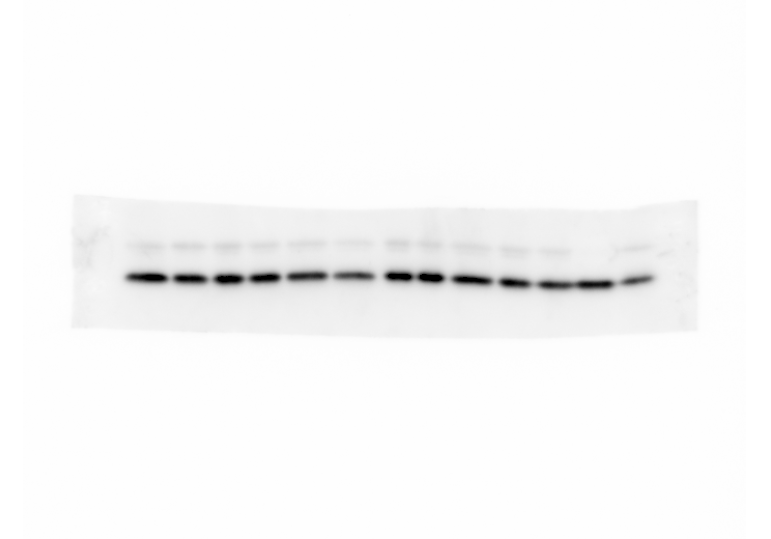


H4 (11 kDa)

1

1

1

1

1

1

1

1

1

1

1

Control

POF

POF+
hEndSCs

POF+
hPMSCs

POF+
hFFSCs

POF+
hMenSCs


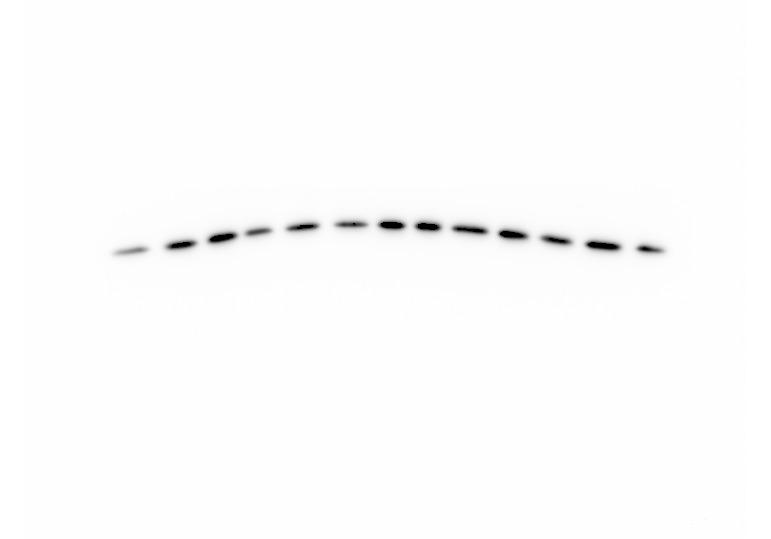

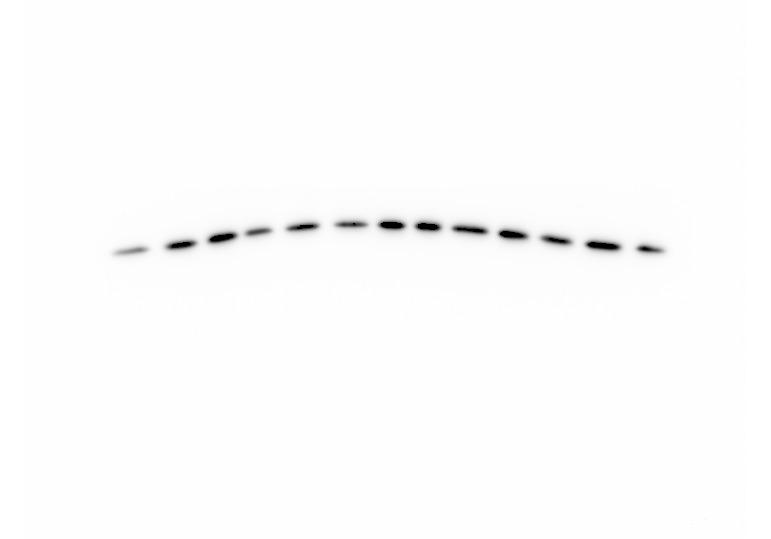


1

1

2

1

2

1

2

1

2

1

2

1

1.9

1.3

1.7

1.5

1.9

1.7

2.2

1.9

1.9

1.6

H4K20me1 (11 kDa)


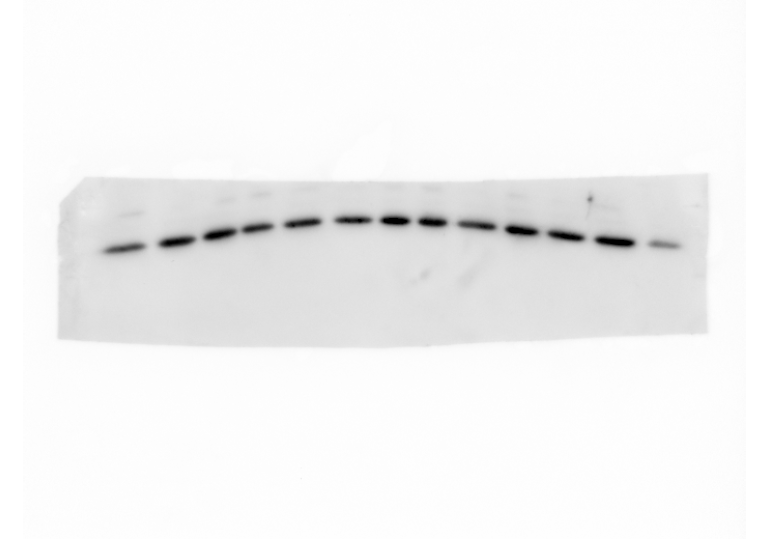

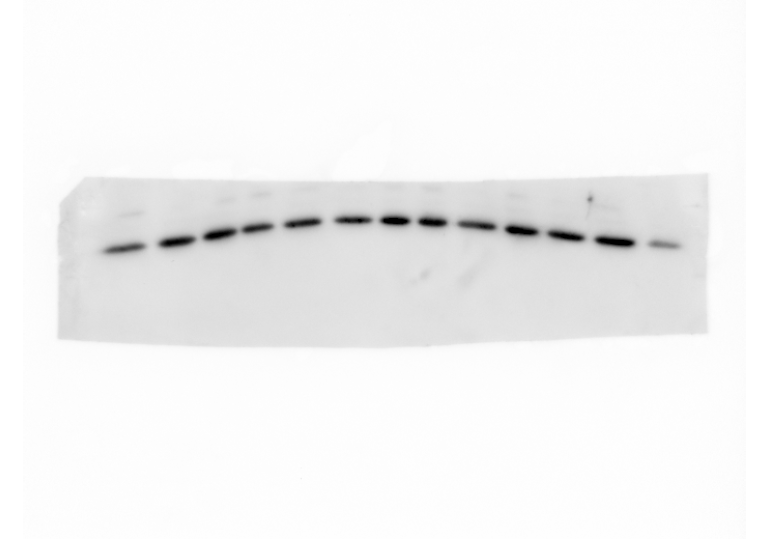


H4 (11 kDa)

1

1

1

1

1

1

1

1

1

1

1

Control

POF

POF+
hEndSCs

POF+
hPMSCs

POF+
hFFSCs

POF+
hMenSCs

1

1

2

1

2

1

2

1

2

1

2


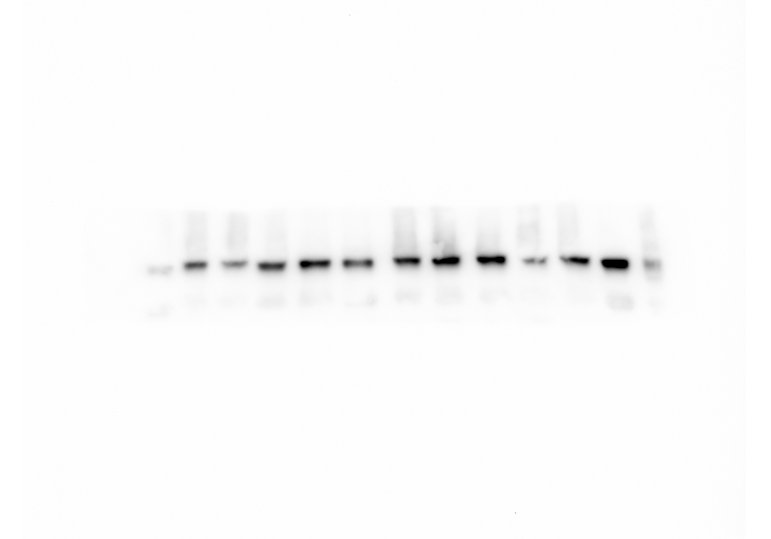

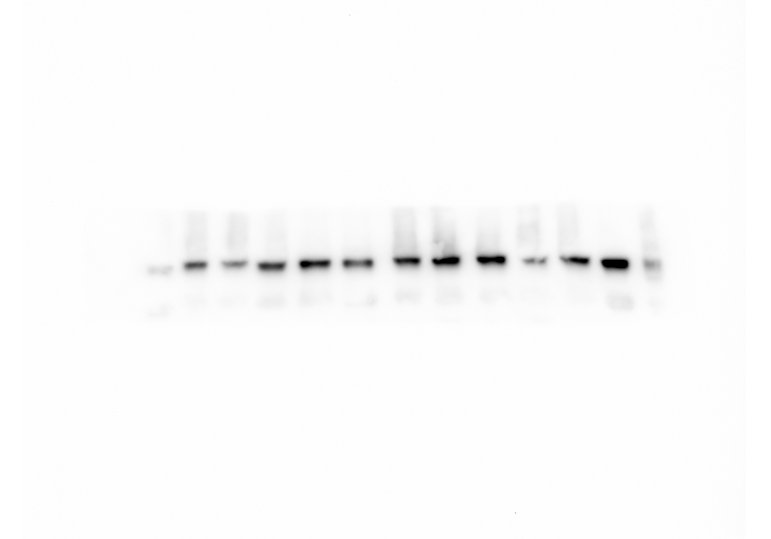


Akt (60 kDa)

Foxo3a
(82-97 kDa)

1

4.3

6.7

13.2

11

11.9

5.6

14.8

3.2

14.7

13.2


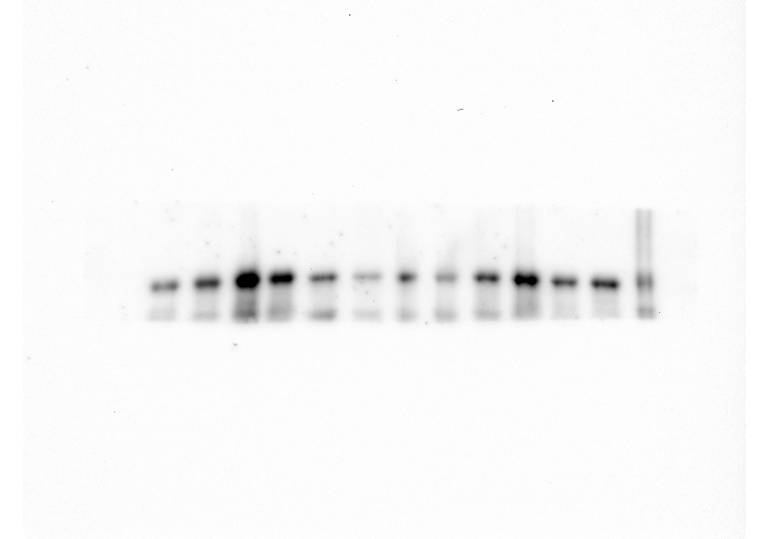

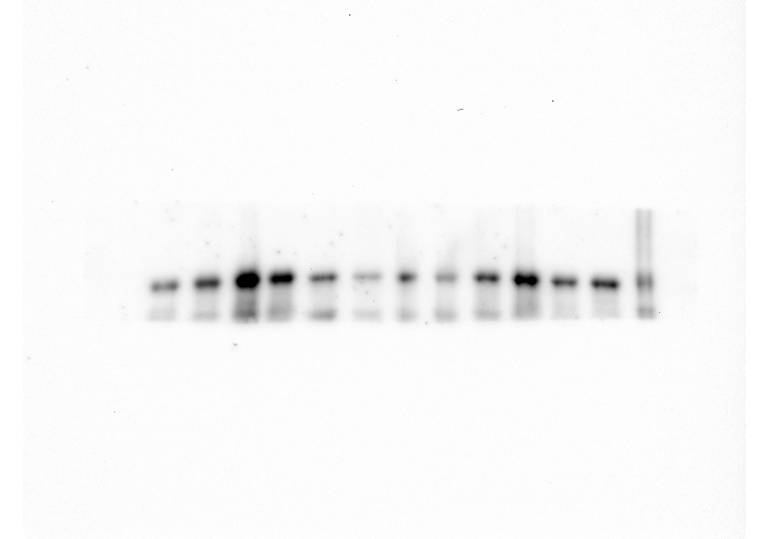


1

3.7

2.3

1.8

0.9

1.7

2.2

2.4

2.8

2.2

3


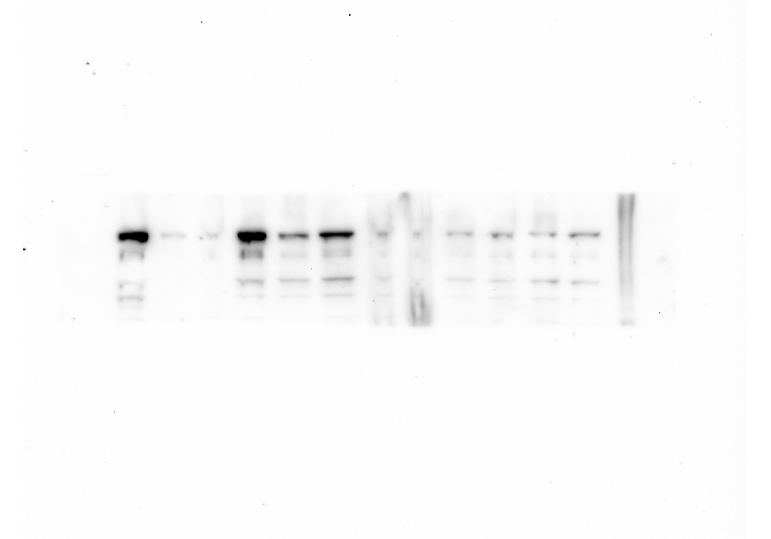

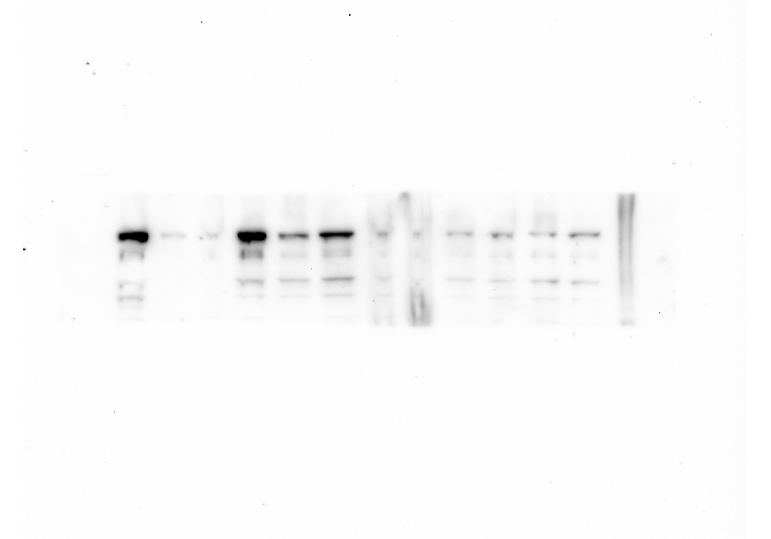


mTOR
 (289 kDa)

1

0.1

1

0.5

1.2

0.2

0.1

0.2

0.2

0.3

0.1


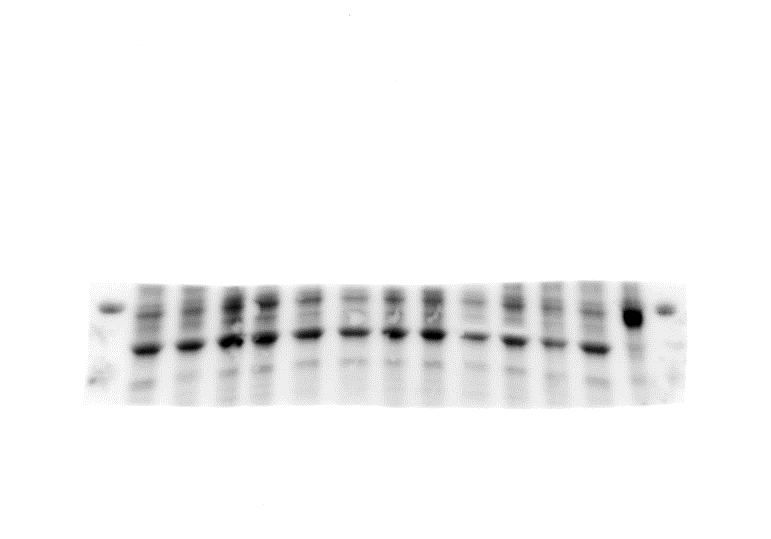

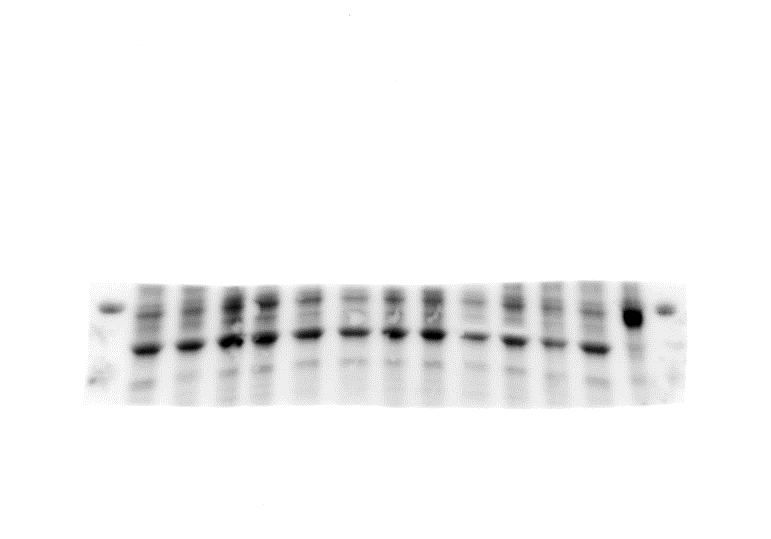


p53 (53 kDa)

1

1.8

1.2

1.6

1.8

1.9

1.5

1.3

1.3

1.4

1.1


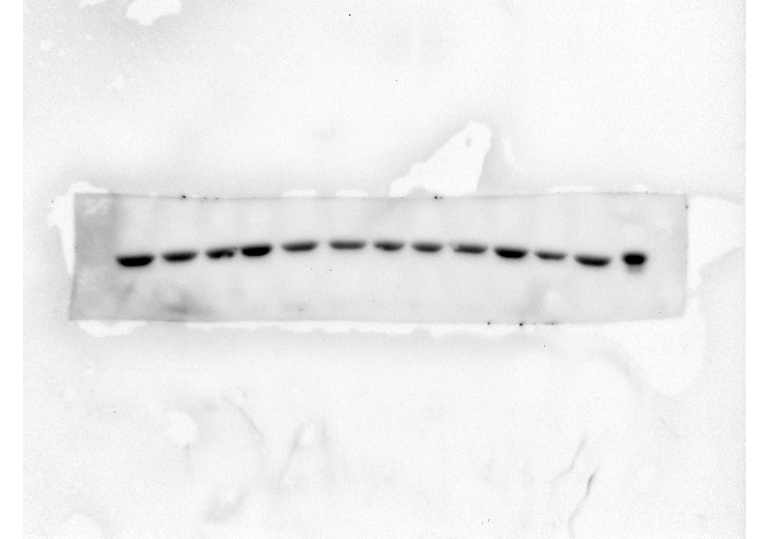

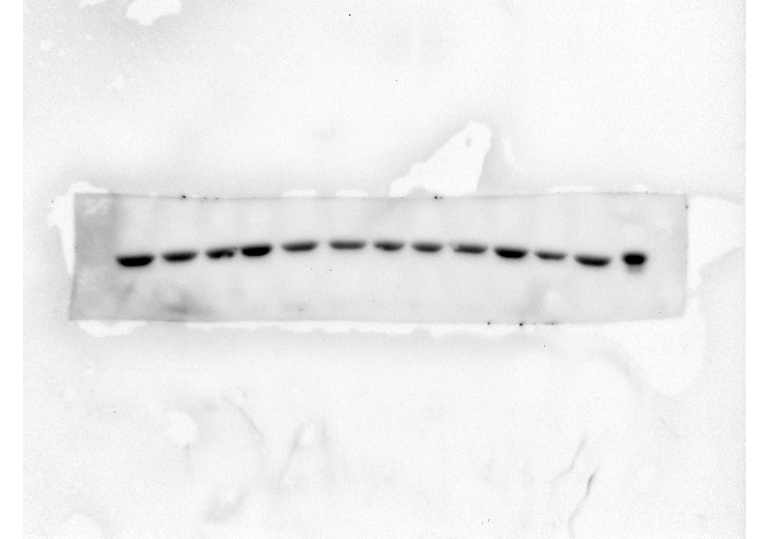


β-actin (42 kDa)

1

1

1

1

1

1

1

1

1

1

1

Control

POF

POF+
hEndSCs

POF+
hPMSCs

POF+
hFFSCs

POF+
hMenSCs

1

1

2

1

2

1

2

1

2

1

2


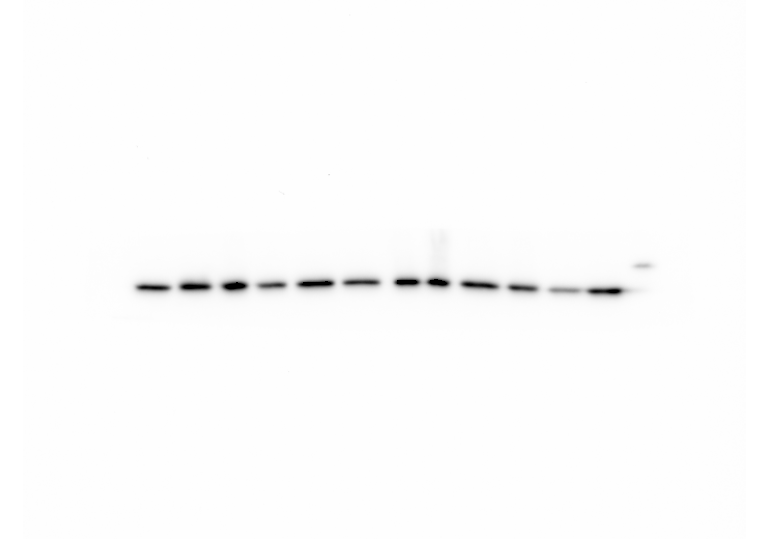

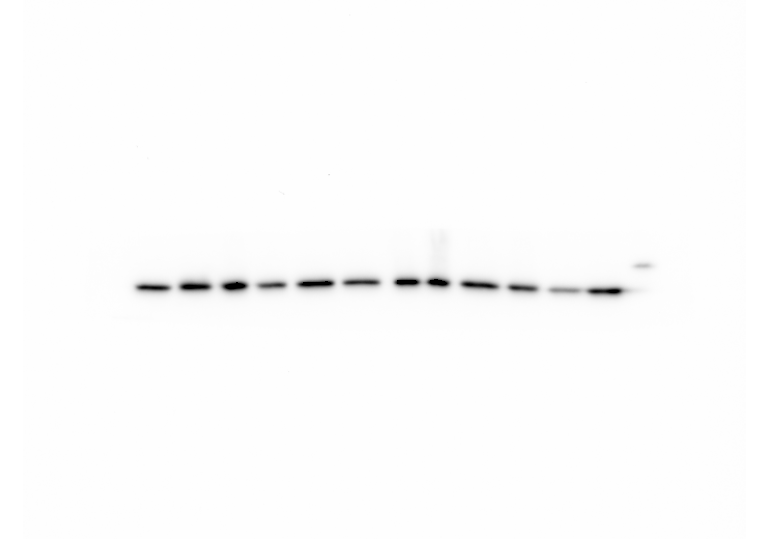


Bcl-2 (26 kDa)

1

1.5

0.7

1.6

1.6

1.6

1.8

1.6

0.8

1.2

0.9


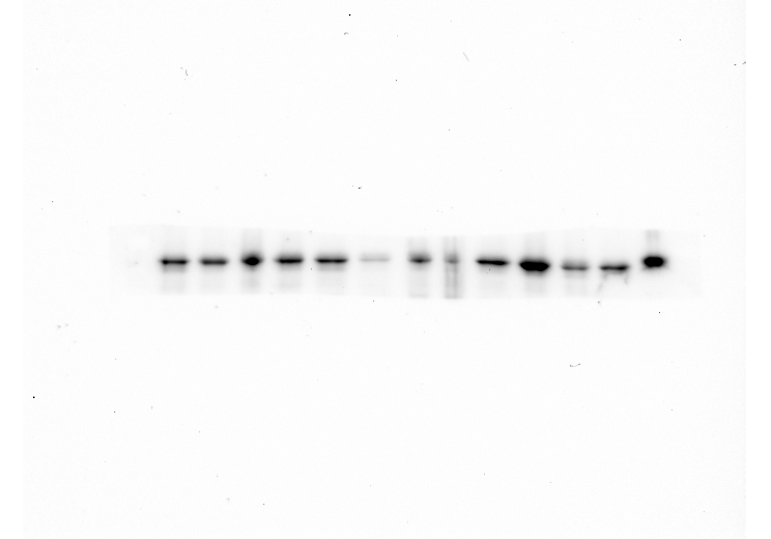


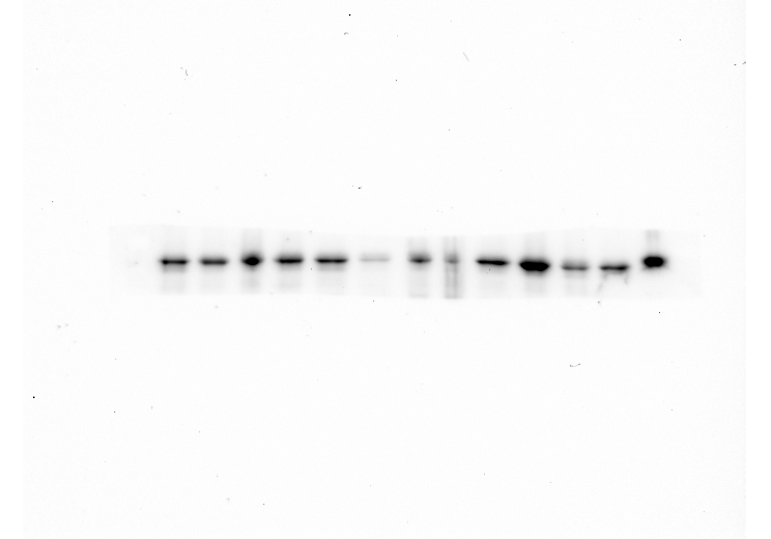


NF-κB (65 kDa)

1

1.3

1

1.3

0.4

1.1

1.2

1.5

1.4

0.8

1


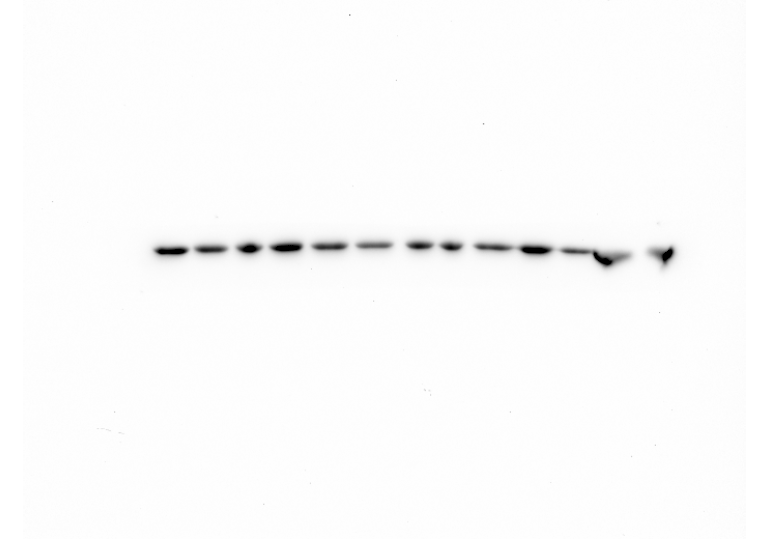

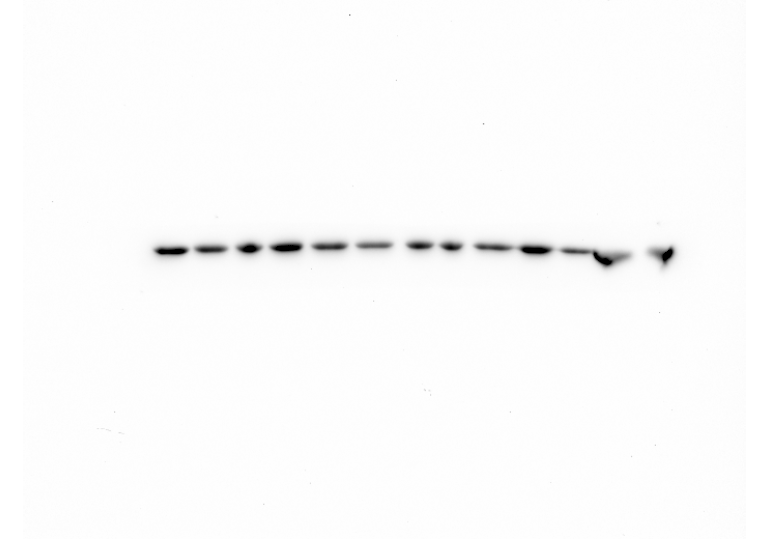


β-actin (42 kDa)

1

1

1

1

1

1

1

1

1

1

1

Control

POF

POF+
hEndSCs

POF+
hPMSCs

POF+
hFFSCs

POF+
hMenSCs

1

1

2

1

2

1

2

1

2

1

2


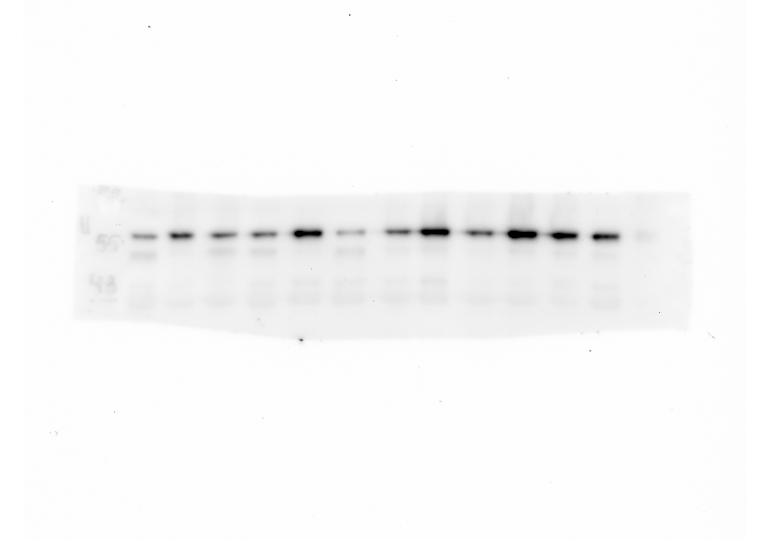

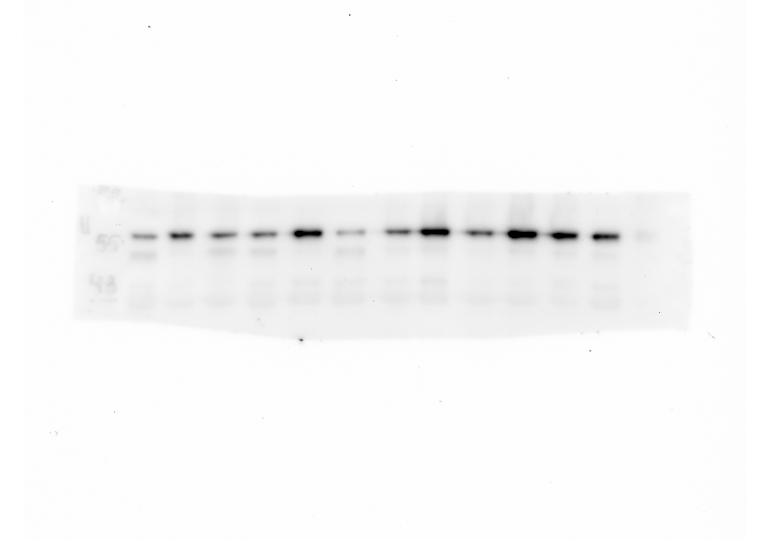


p-Akt (Ser473)
 (60 kDa)

1

1.8

1.4

5

1

2.5

2.3

2.8

6.2

2.9

6


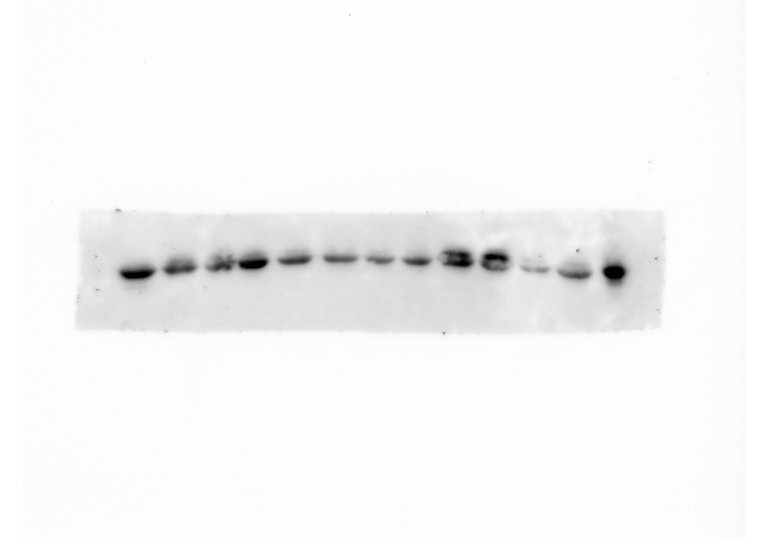

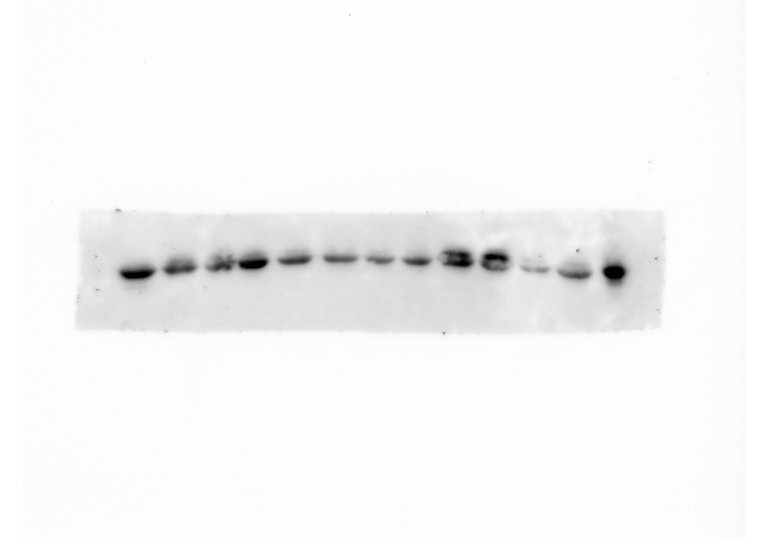


p-GSK-3β (Ser9)
 (46 kDa)

1

1.1

1.2

1

0.8

0.6

1.1

1.9

1.8

0.6

0.5


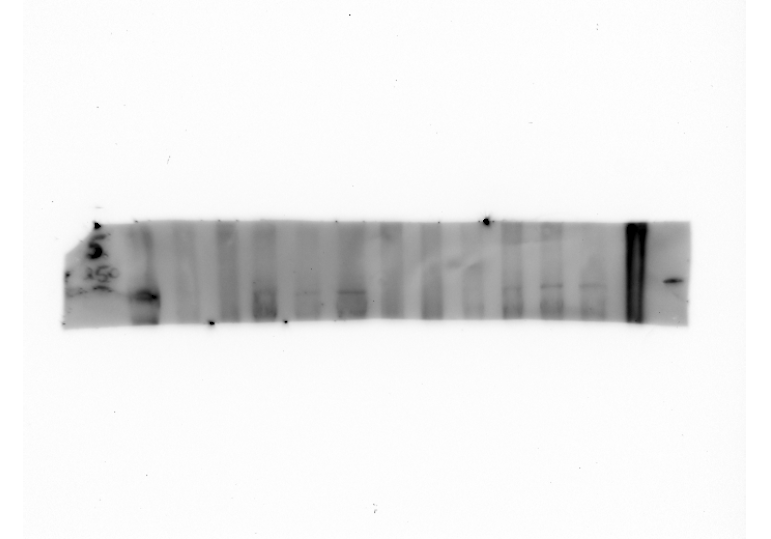

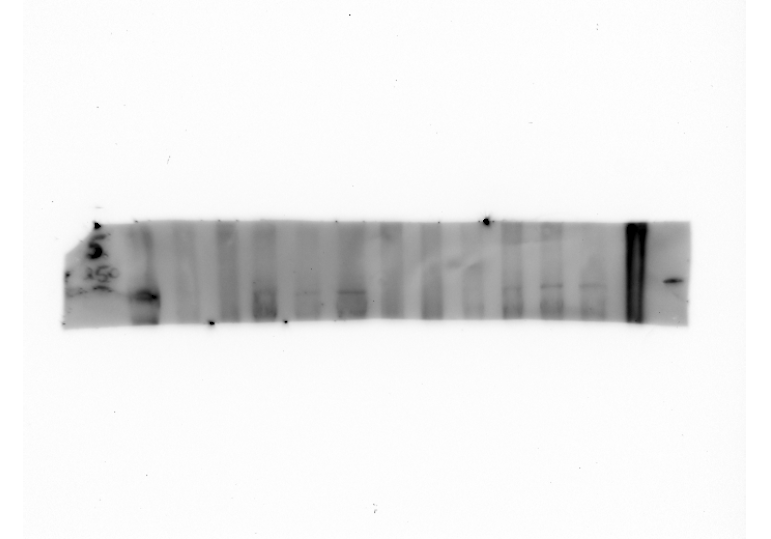


p-mTOR (Ser2448)
 (289 kDa)

1

0

0.03

0.05

0.1

0

0.05

0.01

0.08

0.08

0.1


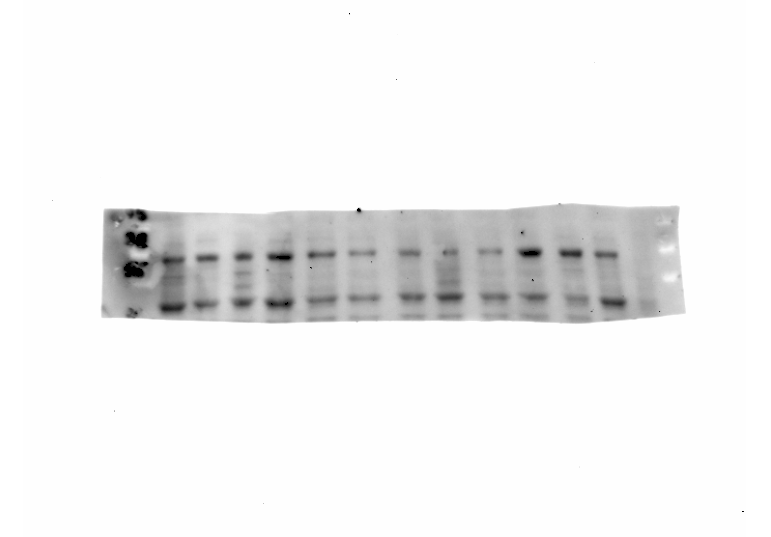

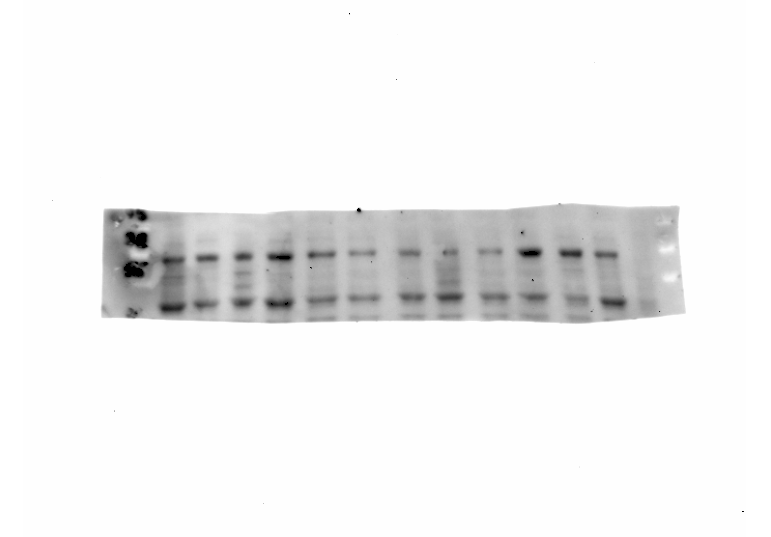


p-p53 (Ser15)
 (53 kDa)

1

2

2.6

2.3

1.6

1.6

2

1.1

4

1.4

4.1


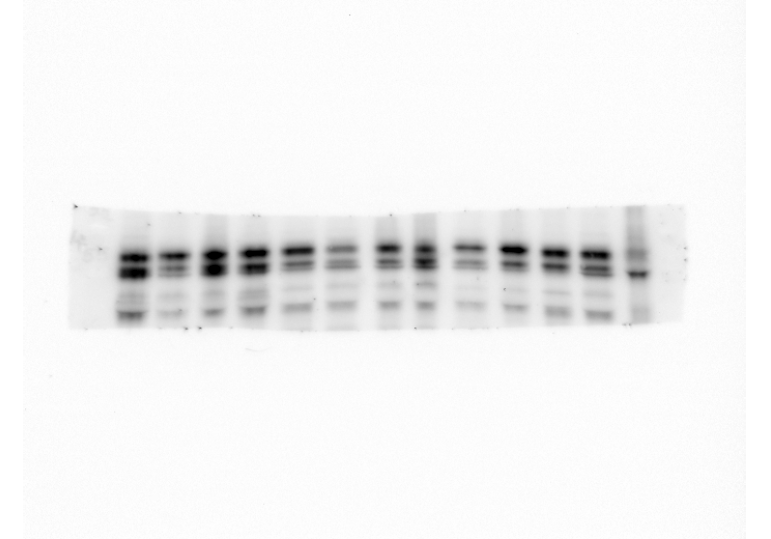


p-PDK1 (Ser241)
 (58-68 kDa)


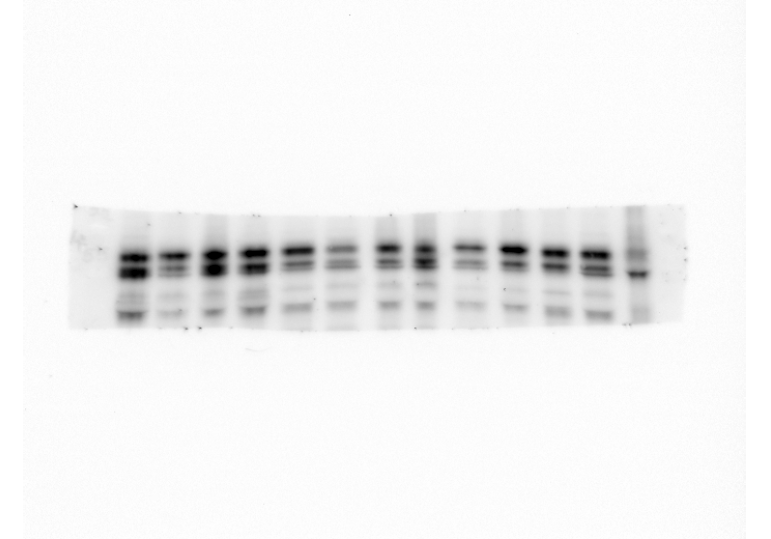


1

1.1

1.6

1

0.6

0.7

0.8

0.6

1

0.9

1.3


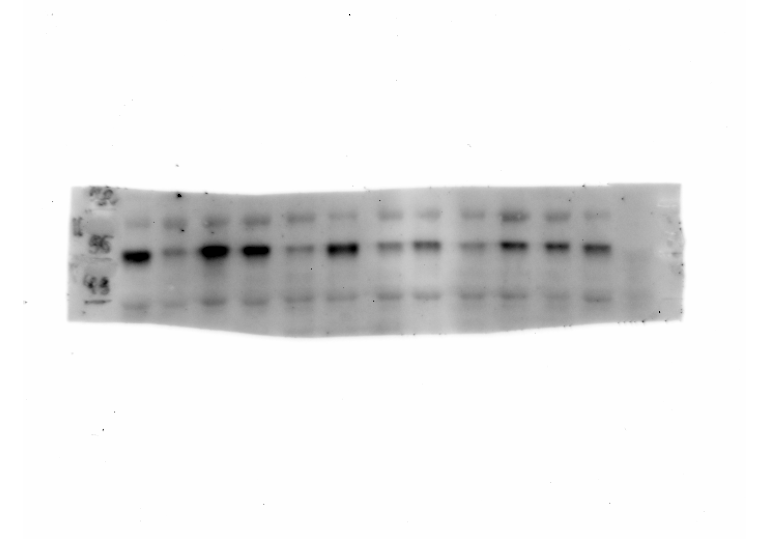

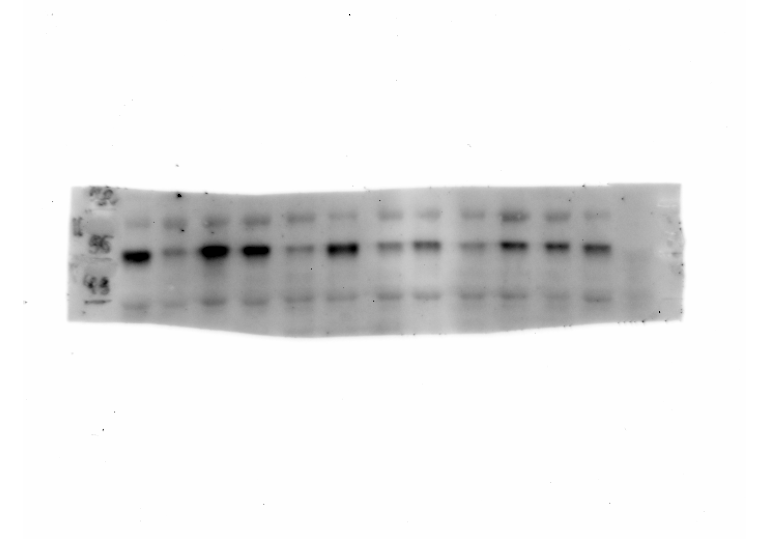


p-PTEN (Ser380)
 (54 kDa)

1

1.2

0.9

0.4

1.3

0.5

0.3

0.4

0.8

0.6

0.9


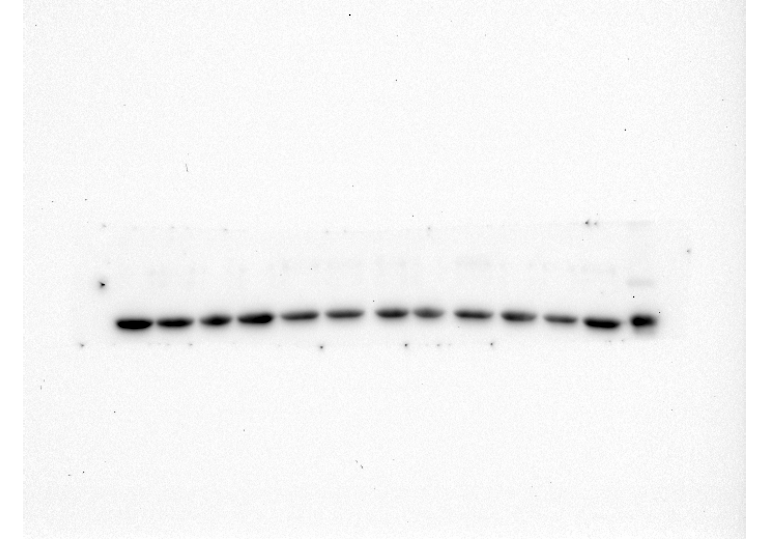

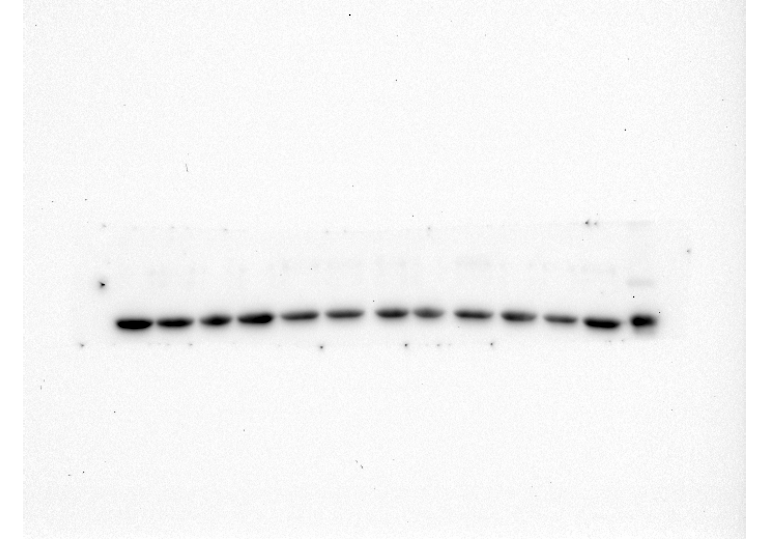


β-actin (42 kDa)

1

1

1

1

1

1

1

1

1

1

1

**Supplementary Figure 2.** Protein bands representing protein levels in mouse uterine tissues. Protein levels associated with epigenetic, cell cycle regulation and endometrial functionality were assessed after premature ovarian failure and stromal cell treatment (hEndSCs, hFFSCs, hMenSCs and hPSCs) using Western blot analysis, n=2. Relative band intensity was calculated using ImageJ software and presented in Figure 9.
